# Infectious blood source alters early foregut infection and regurgitative transmission of *Yersinia pestis* by rodent fleas

**DOI:** 10.1371/journal.ppat.1006859

**Published:** 2018-01-22

**Authors:** David M. Bland, Clayton O. Jarrett, Christopher F. Bosio, B. Joseph Hinnebusch

**Affiliations:** Laboratory of Bacteriology, Rocky Mountain Laboratories, National Institute of Allergy and Infectious Diseases, National Institutes of Health, Hamilton, Montana United States of America; University of Pennsylvania, UNITED STATES

## Abstract

Fleas can transmit *Yersinia pestis* by two mechanisms, early-phase transmission (EPT) and biofilm-dependent transmission (BDT). Transmission efficiency varies among flea species and the results from different studies have not always been consistent. One complicating variable is the species of rodent blood used for the infectious blood meal. To gain insight into the mechanism of EPT and the effect that host blood has on it, fleas were fed bacteremic mouse, rat, guinea pig, or gerbil blood; and the location and characteristics of the infection in the digestive tract and transmissibility of *Y*. *pestis* were assessed 1 to 3 days after infection. Surprisingly, 10–28% of two rodent flea species fed bacteremic rat or guinea pig blood refluxed a portion of the infected blood meal into the esophagus within 24 h of feeding. We term this phenomenon post-infection esophageal reflux (PIER). In contrast, PIER was rarely observed in rodent fleas fed bacteremic mouse or gerbil blood. PIER correlated with the accumulation of a dense mixed aggregate of *Y*. *pestis*, red blood cell stroma, and oxyhemoglobin crystals that filled the proventriculus. At their next feeding, fleas with PIER were 3–25 times more likely to appear partially blocked, with fresh blood retained within the esophagus, than were fleas without PIER. Three days after feeding on bacteremic rat blood, groups of *Oropsylla montana* transmitted significantly more CFU than did groups infected using mouse blood, and this enhanced transmission was biofilm-dependent. Our data support a model in which EPT results from regurgitation of *Y*. *pestis* from a partially obstructed flea foregut and that EPT and BDT can sometimes temporally overlap. The relative insolubility of the hemoglobin of rats and Sciurids and the slower digestion of their blood appears to promote regurgitative transmission, which may be one reason why these rodents are particularly prominent in plague ecology.

## Author summary

*Yersinia pestis*, the bacterial agent of plague, is transmitted by fleas that feed on blood from rodents that carry this disease. The conclusions from studies comparing how efficiently fleas transmit plague after becoming infected have been inconsistent, possibly because a variety of rodent blood sources have been used. To investigate this, we infected three different flea species with *Y*. *pestis* using four different types of rodent blood and compared how well they could transmit three days later. The two rodent flea species that transmitted efficiently tended to reflux bacteria and blood into their esophagus when rat or guinea pig blood was used for the infections, but not when mouse or gerbil blood was used. This reflux phenomenon appears to be related to the solubility of the hemoglobin molecule of different rodent species. In contrast, cat fleas, inefficient transmitters, never refluxed their infected blood meal into the esophagus. Rodent fleas that were infected using reflux-inducing rat blood transmitted more *Y*. *pestis* than those that fed on infected mouse blood. These findings improve our understanding of how fleas transmit *Y*. *pestis* soon after becoming infected and suggest a reason why certain rodents figure more prominently in plague ecology than others.

## Introduction

*Y*. *pestis* is transmitted by the bite of infected fleas, and two modes of transmission have been described: early-phase transmission (EPT) and biofilm-dependent transmission (BDT) [1–3]. Fleas that have taken a highly bacteremic infectious blood meal are capable of EPT on their next feeding attempt within 4 days of becoming infected [4]. An extrinsic incubation period, the time needed for a vector to become infective after acquiring a pathogen, is not required or is very short; fleas can transmit *Y*. *pestis* by 24 h after an infectious blood meal [1]. In contrast, BDT does not typically ensue until at least 5–7 days after infection, the time required for a mature biofilm to form in the proventriculus [5, 6]. The proventriculus is a valve in the flea foregut that regulates the ingress of blood and prevents its backflow into the esophagus [7]. BDT occurs when the *Y*. *pestis* biofilm begins to interfere with or block normal blood feeding. In partially blocked fleas, biofilm accumulation prevents the proventricular valve from closing completely. Blood can still pass through the partially obstructed valve, mix with bacteria in the midgut, and be forced back out through the incompetent valve and into the bite site due to midgut peristalsis and release of pressure from the bloodsucking pump muscles [7, 8]. The biofilm can eventually fill the proventriculus in some fleas, creating a physical barrier to ingestion. When such completely blocked fleas attempt to feed, incoming blood is unable to pass the obstruction, but dissociates bacteria from the biofilm and forces the esophagus to distend [2, 8]. When the flea stops trying to suck blood, elastic recoil of the esophageal wall causes regurgitation of the infectious mixture into the bite site. The physical and molecular mechanisms of BDT are well described; however, neither aspect has been determined for EPT.

The original descriptions of EPT came from experiments in which fleas were fed on a rodent with terminal bacteremia and then collected shortly after their infectious blood meal and transferred individually or in groups to a naïve rodent, which was monitored for plague morbidity and mortality [9–14]. These studies sometimes used different rodent species as the source of the infectious blood meal. When *Oropsylla montana* or *Xenopsylla cheopis* fleas were infected by feeding on bacteremic rats, guinea pigs, or squirrels, 10–100% of animals challenged by groups of 10–100 fleas developed plague [9–11]. However, EPT was rarely observed when individual infected fleas were used (0–1.5% transmission rate per flea), regardless of the flea species or infectious blood source [9, 12–14]. More recent evaluations of early-phase transmission have used an artificial feeder in which rat or mouse blood served as the infectious blood meal. Similar to the earlier studies, when *O*. *montana* or *X*. *cheopis* were infected using rat blood containing ≥10^8^ CFU/ml *Y*. *pestis*, 0–60% and 0–100%, respectively, of Swiss-Webster mice challenged 1 to 4 days later with groups of 7–11 fleas either developed disease or seroconverted, an estimated transmission rate per flea of 0 to ~10% [1, 4, 15, 16]. Fleas apparently transmit few *Y*. *pestis* by the early phase mechanism. In early-phase mass transmission tests in which groups of more than 100 *O*. *montana* or *X*. *cheopis* infected using mouse blood were allowed to feed three days later from a sterile blood reservoir, relatively few CFUs were recovered (median = 3, range = 0–164), and in about half of the experiments no transmission was detected [6]. The total CFUs transmitted in those experiments was below the reported ID50 (250 CFU) of the California ground squirrel, a major host for *O*. *montana* [17].

One variable that may complicate interpretation of disparate vector competence data is the source of rodent blood used in the infectious blood meal [18]. In this study, we evaluated whether different sources of infectious rodent blood (mouse, rat, guinea pig, and gerbil) modulate early infection of the flea foregut by *Y*. *pestis* or EPT. The rodent blood sources used in this study, except for Mongolian gerbil blood, have been previously used in evaluations of flea vector competence for *Y*. *pestis* [1, 2, 6, 10, 12–14]. Gerbil (jird) blood was included in our analysis as *Meriones* spp. are known to be plague reservoir hosts in Asia [19].

We show that ~10–25% of rodent fleas that consumed rat blood or guinea pig blood, but not mouse or gerbil blood, containing high titers of *Y*. *pestis* refluxed a portion of the infectious blood meal from the midgut and proventriculus back into the esophagus shortly after infection, a phenomenon we term PIER (post-infection esophageal reflux). PIER frequently obstructed the passage of blood during the flea’s next blood meal and correlated with increased numbers of *Y*. *pestis* CFU transmitted by *O*. *montana*. Enhanced *in vitro* transmission at 3 days after infection was dependent on the ability of *Y*. *pestis* to produce the exopolysaccharide required for biofilm formation. Thus, when rat blood was used to infect *O*. *montana*, they can become partially or fully blocked and transmit *Y*. *pestis* within a time frame typically attributed to EPT.

## Results

### *X*. *cheopis* fleas often reflux a portion of the blood meal into the esophagus within one day after feeding on certain types of rodent blood containing *Y*. *pestis*

**Fig 1:**
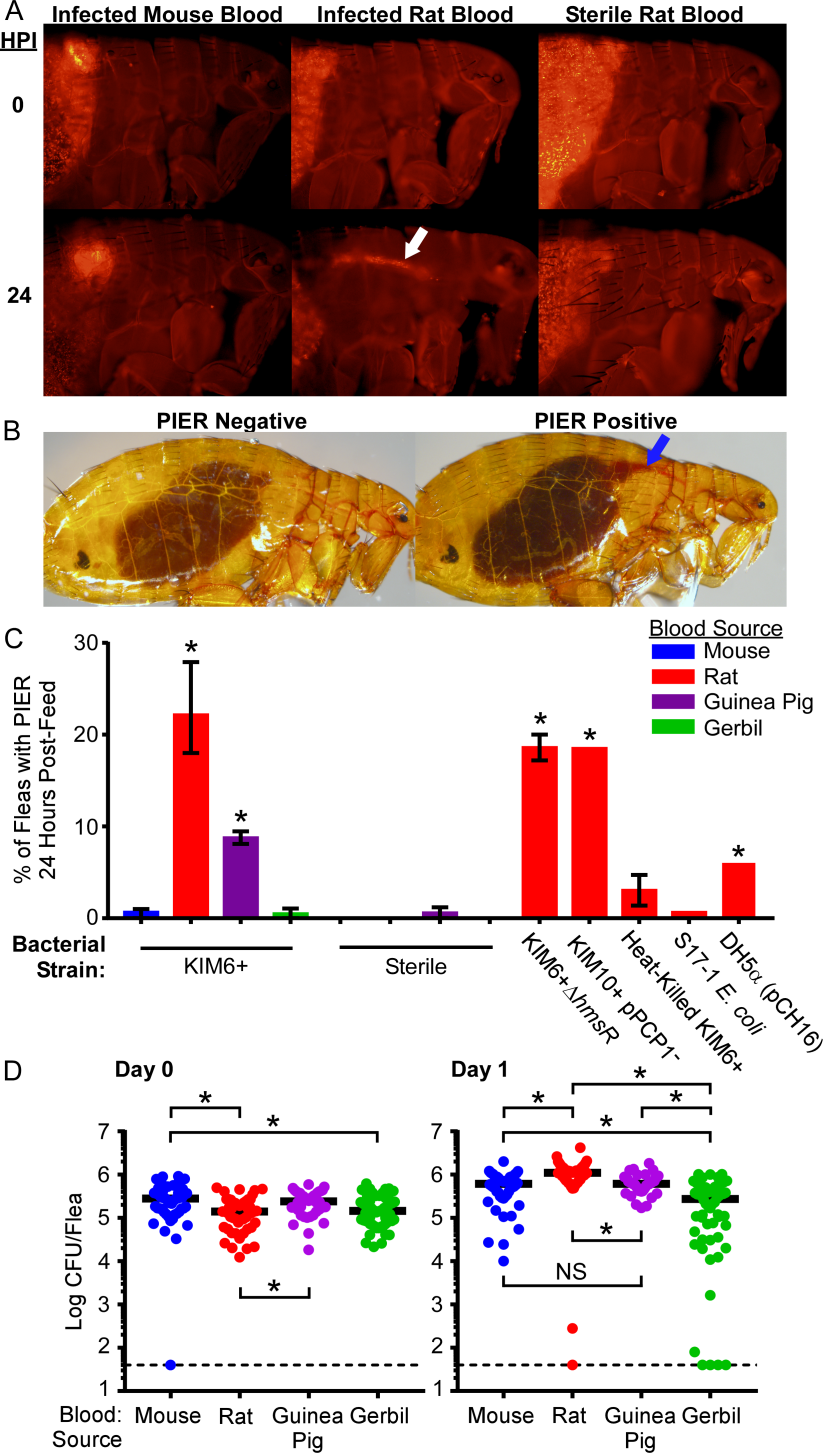
Post-infection esophageal reflux (PIER) occurs in *X*. *cheopis* during the first 24h after infection using rat or guinea pig blood, but not mouse or gerbil blood. (A) Fluorescence microscopy images of *X*. *cheopis* fleas 0 or 24 hours following a sterile or infectious (~1 x 10^9^ CFU/ml KIM6+pAcGFP1 *Y*. *pestis*) rat or mouse blood meal supplemented with 1 μm red fluorescent beads. White arrow indicates the fluorescent beads in the esophagus. (B) Examples of fleas with and without PIER. Blue arrow indicates the fresh regurgitated blood meal in the esophagus that is diagnostic for PIER. (C) Incidence of PIER in *X*. *cheopis* fleas 24 h after they had fed on mouse, rat, guinea pig, or gerbil blood containing 3.3 x 10^8^–2 x 10^9^ CFU/ml live or heat-killed *Y*. *pestis* KIM6+ (pAcGFP1), KIM6+ Δ*hmsR* (pAcGFP1), KIM10+ (pAcGFP1), *E*. *coli* S17-1, or *E*. *coli* DH5α (pCH16); or on sterile blood of the rodent species. The cumulative mean and range of 1–4 independent experiments (n = 121–689 fleas) are shown. *p < 0.001 compared to infectious mouse blood group by chi-square test with Bonferonni post-test. (D) Bacterial load of individual infected female *X*. *cheopis* on day 0 or day 1 after infection. *p < 0.05 (day 0) or p < 0.01 (day 1) by Kruskal-Wallis test with Dunn’s post-test. n = 40–60 female fleas from 2–3 independent experiments. Horizontal bars represent the median. Limit of detection (dashed line) = 40 CFU.

When a flea feeds, muscles located in the head pump blood through the esophagus, the proventriculus, and into the midgut [20]. As feeding ends, the esophagus is cleared of residual blood while the proventriculus and hindgut confine the blood meal while it is digested in the midgut [21]. Unexpectedly, 24 h after *X*. *cheopis* fleas consumed rat blood containing ~1 x 10^9^ *Y*. *pestis* KIM6+ (pAcGFP1) CFU/ml and 1 μm fluorescent beads, both blood and beads were observed in the esophagus in 21 of 80 (26%) of fleas (Fig 1A). We termed this post-infection esophageal reflux (PIER). The distance blood and beads refluxed through the esophagus due to PIER was variable, ranging from just above the proventricular-esophageal junction to within the head. In contrast, PIER was not observed when fleas were fed sterile rat blood or when mouse blood served as the infectious blood meal; the blood meal remained confined to the proventriculus and MG. Because PIER was not evident immediately after the infectious blood meal and the fleas had not fed subsequently, we concluded that a portion of the blood meal had refluxed from the proventriculus or MG back into the esophagus during the first 24 h of infection.

To determine the incidence of PIER in infected flea populations and confirm that the fluorescent beads did not induce PIER, groups of 82–296 rat fleas were fed a sterile or high-titer infectious blood meal (without fluorescent beads) from 1 of 4 different rodent species: Sprague-Dawley rat (*Rattus norvegicus*), RML Swiss-Webster mouse (*Mus musculus*), domestic guinea pig (*Cavia porcellus*), or Mongolian gerbil (*Meriones unguiculatus*). Immediately following feeding, and again 24 h later, fleas were immobilized, examined under a microscope, and the number of fleas with and without PIER was recorded (Fig 1B). One day after feeding on infected rat or guinea pig blood, 8–28% of fleas exhibited PIER (Fig 1C). In contrast, ≤1.2% of fleas fed bacteremic mouse or gerbil blood, or fleas fed sterile blood from any rodent species, developed PIER (Fig 1C). Induction of PIER did not require the *Y*. *pestis hmsHFRS*-dependent exopolysaccharide needed for biofilm formation, or the Pla protease (Fig 1C and Table 1). Adding live *Escherichia coli* S17-1 or DH5α (pCH16), or heat-killed *Y*. *pestis* to the blood meal did not induce PIER at rates comparable to live *Y*. *pestis* (0–6% vs. ~22%, respectively), indicating that metabolic activity and/or replication of *Y*. *pestis* in the digestive tract enhances development of PIER.

**Table 1:** Bacterial strain and plasmid list.

Strain/Plasmid	Key Properties	Reference
***Y*. *pestis* strains**		
KIM6+	pCD1^-^, pMT1+, pPCP1+, Pgm+	[22, 23]
KIM6+Δ*hmsR*	KIM6+ deleted of the glycosyltransferase gene *hmsR* that is required for biofilm formation, flea blockage, and pigmentation on Congo red agar.	This Study
KIM10+	pCD1^-^, pMT1+, pPCP1^-^, Pgm+, lacks the gene required to produce the Pla protease	[24, 25]
***E*. *coli* strains**		
DH5α	Cloning strain	[26]
S17-1	Cloning strain, λ*pir*+	[27]
**Plasmids**		
pAcGFP1	Ap^r^, constitutively expresses GFP	Clontech, (Mountain View, CA)
pCH16	Ap^r^, Km^r^, constitutively expresses Yersinia murine toxin (Ymt), a phospholipase D enzyme important for bacterial colonization of the flea midgut.	[28]
pCVD442*hmsR*	Ap^r^, suicide vector used to generate KIM6+Δ*hmsR*	This Study, [29]
pDONR221*hmsR*	Km^r^, Gateway cloning vector containing *hmsR* ORF from *Y*. *pestis* KIM	Pathogen Functional Genomics Resource Center, J. Craig Venter Institute

pCD1 virulence plasmid, encodes type 3 secretion system

pMT1 plasmid, encodes the phospholipase D Yersinia murine toxin (Ymt) and capsule antigen (F1)

pPCP1 plasmid, encodes plasminogen activator/protease (Pla), the bacteriocin pesticin (Pst), and pesticin immunity protein (Pim)

Pgm pigmentation locus and pathogenicity island, encodes the hemin storage locus (*hmsHFRS* operon) and iron acquisition genes

Ap^r^ ampicillin resistance; Km^r^ kanamycin resistance

To verify that the method of anti-coagulation did not influence induction of PIER, fleas were infected with both defibrinated and sodium heparin treated mouse or rat blood (Fig 1C and S1 Fig). Identical rates of PIER were observed in infected fleas regardless of the treatment used to prevent the blood from clotting. Furthermore, PIER occurred in fleas infected using washed rat RBCs in PBS, indicating that coagulation factors or other plasma components are not essential for PIER (S1 Fig).

The data show that a portion of the infectious blood meal is often refluxed into the esophagus within 24 h of ingesting rat or guinea pig blood containing high titers of *Y*. *pestis*, but not when bacteremic mouse or gerbil blood is ingested.

### Blood-related differences in PIER induction are not attributable to differences in flea infection rate or bacterial burden

A previous study indicated that the species of host blood affects *Y*. *pestis* infection rates in rodent fleas [18]. We found that in the short term (1 day after infection), *X*. *cheopis* that fed on blood containing ~5 x 10^8^ *Y*. *pestis*/ml had similar infection rates and bacterial loads regardless of the rodent blood used for the infectious blood meal (Fig 1D). Flea groups infected with mouse, rat, or guinea-pig blood had 100% infection rates and equivalent numbers of *Y*. *pestis* (~5 x 10^5^–1 x 10^6^ CFU/flea) in the digestive tract for 24 h following the infectious blood meal. Fleas infected using gerbil blood had slightly lower infection rates (93%) and more variable bacterial burdens; however, the majority had bacterial burdens comparable to those infected using other rodent blood sources. These results indicate that differences in replication or clearance of *Y*. *pestis* in the flea digestive tract do not explain why some blood sources induce PIER while others do not.

### PIER correlates with a more viscid colonization of the proventriculus and localization of *Y*. *pestis* to the esophagus

**Fig 2:**
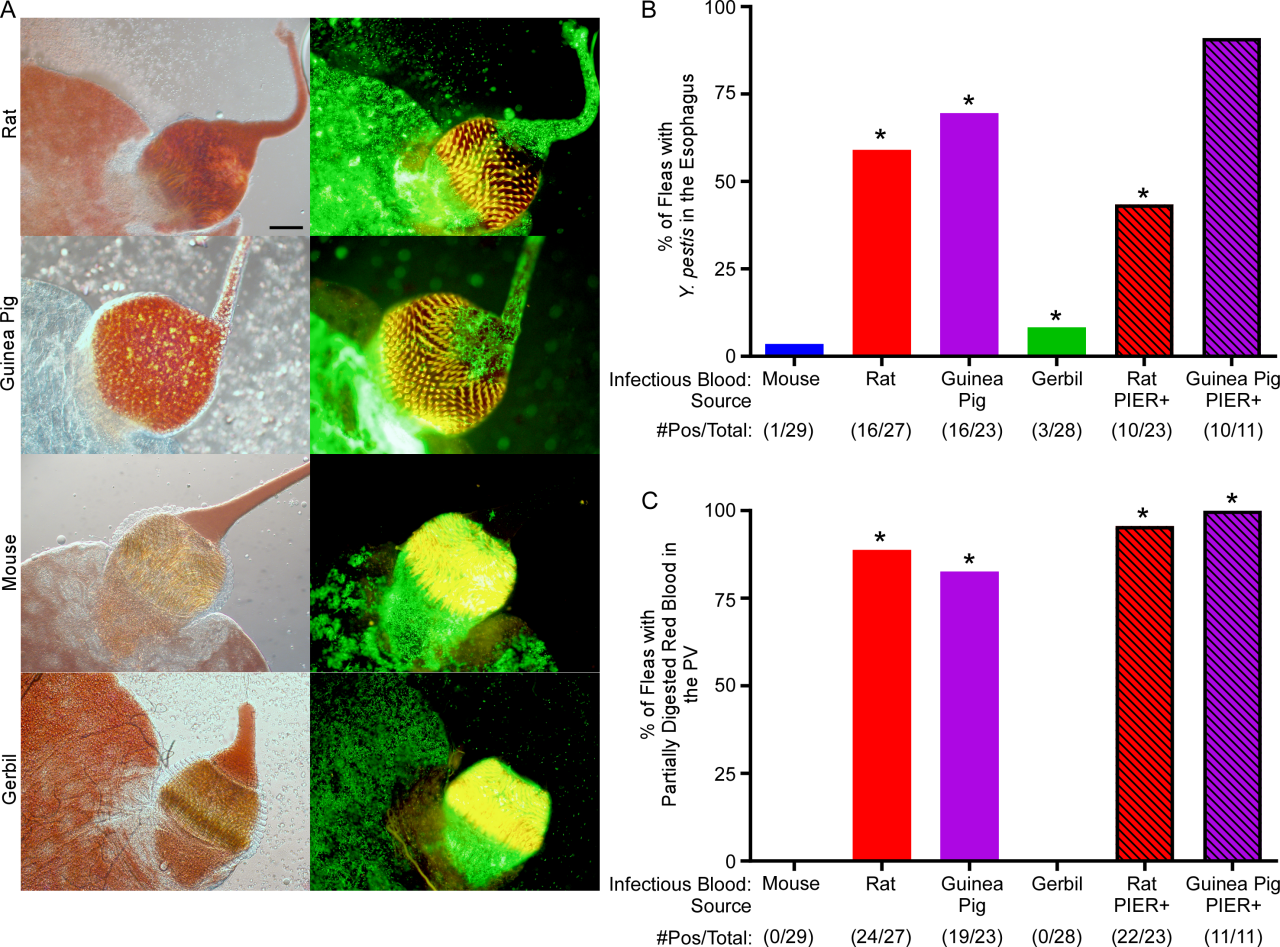
*Y*. *pestis* frequently localizes to the esophagus of *X*. *cheopis* following an infectious blood meal of rat or guinea pig blood. (A) 11–29 mixed-sex *X*. *cheopis* fleas (with or without PIER) were dissected 24 hours after ingesting infected blood from 1 of the 4 rodent sources. Digestive tracts were scored for the presence or absence of GFP+ bacteria in the esophagus and partially digested red blood in the proventriculus that obscured visualization of the proventricular spines. Representative bright field (left) and fluorescence (right) microscopy images of dissected *X*. *cheopis* foreguts are shown. (B) Percentage of fleas with *Y*. *pestis* (GFP+) in the esophagus; (C) Percentage of fleas with incompletely digested blood meal material in the proventriculus that obscured visualization of the proventricular spines. Proventricular spines autofluoresce yellow-orange. Numbers below the x-axis indicate the number of fleas positive/the total number of infected fleas dissected. The cumulative mean of 2–4 independent experiments are shown. *p < 0.001 compared to infectious mouse blood group by chi-square test with Bonferonni post-test. Scale bar = 50 μm.

*Y*. *pestis* is non-motile and does not adhere to the midgut epithelium during the early stages of flea infection, so it seemed likely that plague bacilli would also be present in the esophagus following PIER [30]. To confirm this, the digestive tract of *X*. *cheopis* fleas were scored for PIER 24 h after infection, dissected, and the foregut was screened for localization of GFP+ bacteria to the esophagus. Digestive tracts of fleas infected with rat or guinea pig blood containing *Y*. *pestis* had high rates (~40–90%) of bacterial localization to the esophagus (Fig 2A and 2B). Conversely, only 3 to 10% of fleas infected using mouse or gerbil blood contained *Y*. *pestis* in the esophagus, even though 88% (50 of 57) of them contained *Y*. *pestis* in the proventriculus.

Initially, the proventriculus of all fleas contained a portion of the blood meal regardless of host blood source. Placement of a coverslip on the dissected digestive tract of all 57 fleas infected using mouse or gerbil blood was sufficient to force most this out into the esophagus or back into the midgut, leaving the valve somewhat flattened and its spines clearly distinct (Fig 2A). In contrast, the proventriculus of 43 of 50 fleas (86%) infected using rat or guinea pig blood, and of 33 of 34 fleas diagnosed with PIER, remained bulbous and retained blood meal contents, which obscured visualization of the proventricular spines (Fig 2A and 2C). In addition, the proventricular spines of fleas infected with rat or guinea pig blood appeared to be spread further apart when compared to spines of fleas infected using mouse or gerbil blood (Fig 2A). Thus, the flea foregut is rapidly colonized following the infectious blood meal and certain rodent blood sources promote PIER, esophageal localization of *Y*. *pestis*, and enhanced retention of partially digested infected blood in the proventriculus.

### PIER correlates with accumulation of partially digested RBCs and oxyhemoglobin crystals in the flea digestive tract

**Fig 3:**
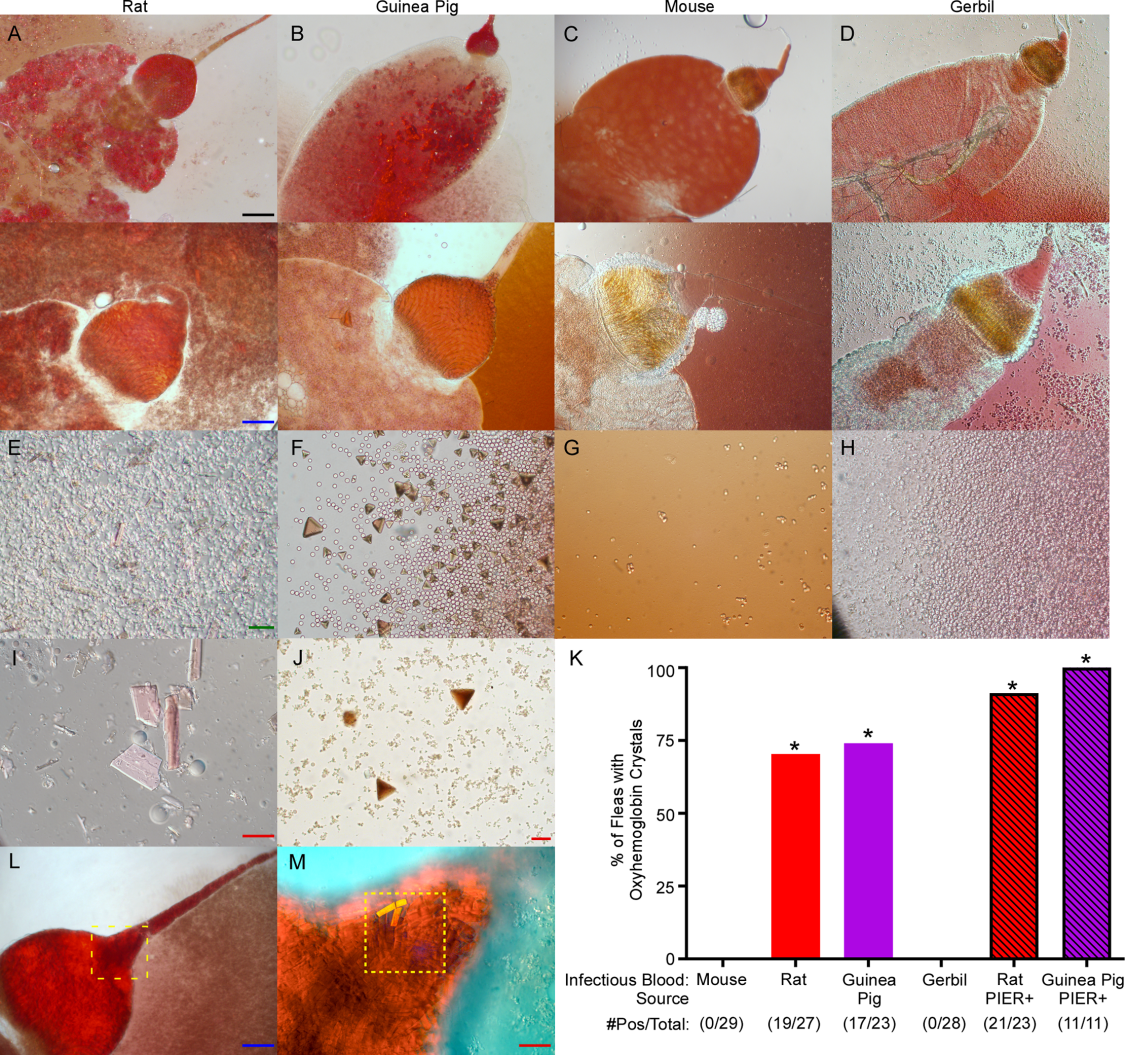
Numerous oxyhemoglobin crystals form in the digestive tract of fleas infected using a PIER-inducing blood source. Digestive tracts (A-D) and midgut contents (E-J) dissected from *X*. *cheopis* 24h after they had fed on infected rat (A, E, I), guinea pig (B, F, J), mouse (C, G) or gerbil (D, H) blood. Partially digested red blood cell stroma and rhomboid to trapezoid-shaped or rhomboid-shaped crystals were abundant following rat or guinea pig blood meals, respectively. Images are representative of 11–29 dissections. (K) Percentage of total and PIER+ *X*. *cheopis* digestive tracts containing oxyhemoglobin crystals one day after feeding on infected blood from one of the four rodent sources. The cumulative mean from 2–4 independent experiments are shown; the number of fleas positive/ total number of fleas dissected is indicated below the *x*-axis. *p < 0.0001 compared to infectious mouse blood group by chi-square test with Bonferonni post-test. (L) Example of a flea with PIER (infected with rat blood); and (M) a higher magnification image of the proventricular-esophageal junction (dashed-yellow boxed area) containing numerous rhomboid oxyhemoglobin crystals. Three oxyhemoglobin crystals are highlighted in yellow. Image contrast, color balance, and tone were modified for image (M) to more easily visualize oxyhemoglobin crystals. Scale bars: black = 100 μm, blue = 50 μm, green = 20 μm, red = 10 μm.

Upon dissection of *X*. *cheopis* that fed on rat or guinea pig blood, the presence of large numbers of crystals in the digestive tract became apparent (Fig 3A, 3B, 3E, 3F, 3I and 3J). These crystals were not observed in the digestive tract of infected fleas that fed on mouse or gerbil blood (Fig 3C, 3D, 3G and 3H). The morphology and color of these crystals were identical to descriptions of oxyhemoglobin crystals of the rat (rhomboidal or trapezoidal) and guinea pig (pyramidal) [31, 32]. Most of the rat hemoglobin crystals in the flea guts formed rhomboids, but some were jagged and trapezoidal. Crystallization of oxyhemoglobin during digestion of sterile rat and guinea pig blood has been observed previously in the gut of several blood-feeding arthropods, including soft ticks, kissing bugs, lice, and *X*. *cheopis* fleas [33–36]. Consistent with previous observations, oxyhemoglobin crystal formation was not dependent on the presence of *Y*. *pestis*: all 10 fleas dissected 4 h after feeding on sterile rat blood contained numerous crystals in the gut. Crystals varied in size and concentration but were present in >70% of fleas fed rat or guinea pig blood containing *Y*. *pestis* for at least 24 h following the infectious blood meal (Fig 3K). Oxyhemoglobin crystals were prevalent in the midgut of 32 of 34 fleas diagnosed with PIER and for many of these, crystals were also observed in the proventricular-esophageal junction (Fig 3L and 3M). Even though the thick muscle layers surrounding the proventriculus precluded direct observation of crystals, the general abundance of oxyhemoglobin crystals in the gut and frequent observations of oxyhemoglobin crystals within the proventricular-esophageal junction and esophagus indicate that the material in the proventriculus also included oxyhemoglobin crystals.

*Rattus norvegicus* hemoglobin is poorly soluble and readily crystalizes after RBC hemolysis [32, 37, 38]. We generated hemoglobin crystals with similar morphology to those found in the flea gut by lysing rat RBCs in sterile water at room temperature for 10 minutes (S2A Fig). Lysis of mouse, guinea pig, or gerbil RBCs using an identical protocol did not result in the formation of hemoglobin crystals. Absorption spectra of a suspension of the crystals derived from rat RBCs showed peaks at 540 and 570 nm, which is characteristic of oxyhemoglobin (S2B Fig) [33, 37].

Rodent blood sources with poorly soluble hemoglobin were also digested more slowly in the flea gut than was mouse blood. Most fleas infected with rat, guinea pig, or gerbil blood contained abundant partially digested RBC stroma 24 h after infection, whereas most fleas that consumed infectious mouse blood had completely digested their blood meal–the gut primarily contained a clear, viscous sepia-colored liquid and little or no RBC stroma (3E-H).

To directly assess whether rat RBCs are critical for induction of PIER, *X*. *cheopis* fleas were infected with *Y*. *pestis* suspended in washed rat RBCs in PBS (S1 Fig). PIER was seen in 15% of these fleas, indicating that rat RBCs alone are sufficient to induce PIER. Furthermore, addition of lysed rat RBC components (stroma and oxyhemoglobin crystals) to infectious mouse blood (which would not normally induce PIER) was sufficient to cause PIER in some fleas that fed on the mixture (S1 Fig). PIER was only observed when rat RBCs were present in the infectious blood meal.

The observations collectively indicate that the proventriculus becomes congested with a combination of oxyhemoglobin crystals, bacteria, and RBC stroma when fleas feed on rat or guinea pig blood containing *Y*. *pestis*.

### PIER occurs in efficient vectors of *Y*. *pestis*

**Fig 4:**
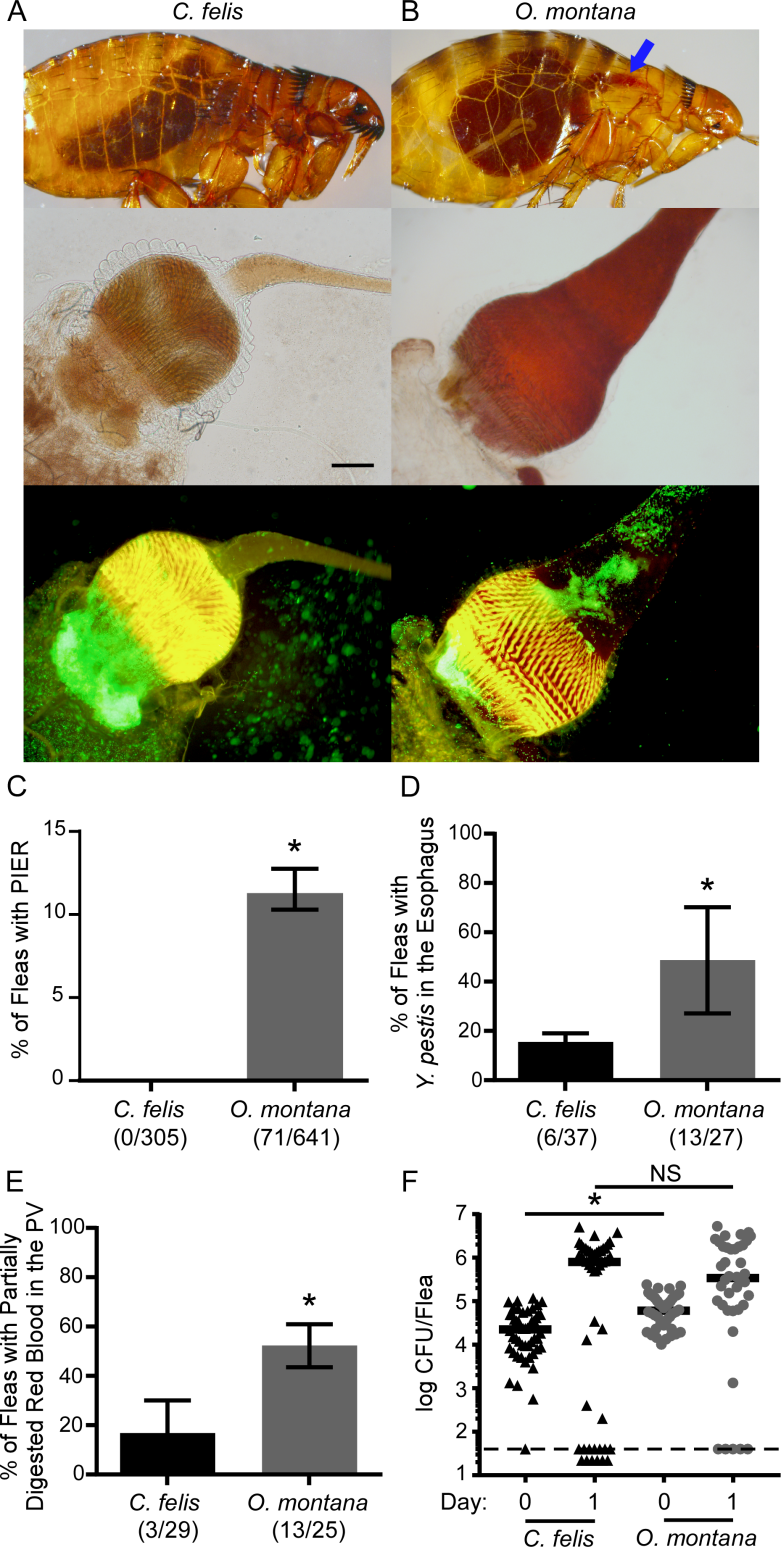
PIER regularly occurs in infected *O*. *montana* fleas, but not in *C*. *felis*. Groups of 85–204 mixed-sex *C*. *felis* or *O*. *montana* were fed rat blood containing 3.3 x 10^8^−1.2 x 10^9^ *Y*. *pestis* KIM6+ (pAcGFP1) CFU/ml. 1 day after infection, fleas were screened for PIER and dissected for evidence of *Y*. *pestis* in the esophagus and accumulation of partially digested red blood in the foregut. (A) Representative examples of an infected *C*. *felis* without PIER and the foregut dissected from an infected *C*. *felis*. (B) Examples of intact and dissected *O*. *montana* fleas with PIER (blue arrows indicate the fresh regurgitated blood in the esophagus). (C) Percentage of infected *C*. *felis* (black columns) and *O*. *montana* (grey columns) with PIER, (D) GFP+ bacteria in the esophagus, or (E) bright red blood in the proventriculus that obscured visualization of the proventricular spines. The cumulative mean and range of 3–5 independent experiments is shown; the number of fleas positive/total number of infected fleas dissected and/or screened is indicated below the *x*-axis. (F) Bacterial load of individual infected female fleas on day 0 or day 1 of infection from 3 independent experiments (n = 40–50). Black and grey symbols indicate results for *C*. *felis* and *O*. *montana*, respectively; horizontal bars indicate the median. For PIER diagnosis, GFP+ bacteria in the esophagus, and blood in the proventriculus, *p < 0.01 by chi-squared test. For bacterial loads, *p < 0.0001 or NS = no significant difference by Mann-Whitney test. Limit of detection (dashed line) = 40 CFU. Scale bar = 50 μm.

To determine whether other known vectors of *Y*. *pestis* develop PIER, we infected groups of *O*. *montana* (like *X*. *cheopis*, an efficient EPT vector) and *C*. *felis* (a poor EPT vector) using rat blood and screened them for PIER. PIER was not seen in infected *C*. *felis* 24 h after the infectious blood meal (Fig 4A and 4C). In contrast, PIER was observed in 11% of *O*. *montana*, an incidence rate that was about half that observed for infected *X*. *cheopis* (Figs 1C, 4B and 4C). Similarly to *X*. *cheopis*, ~50% of infected *O*. *montana* had bacteria localized to the esophagus and accumulated partially digested blood debris in the foregut, which frequently obscured visualization of the proventricular spines (Fig 4B, 4D and 4E). The percentage of infected *C*. *felis* with bacteria in the esophagus and partially digested red blood in the foregut was significantly lower, 16 and 10%, respectively. 80–95% of *O*. *montana* remained infected 24 h following the infectious blood meal whereas the *C*. *felis* infection rate was more variable, ranging from 50–100% (Fig 4F). Bacterial burdens were comparable between flea species. Oxyhemoglobin crystals were observed in the digestive tract of both flea species; however, they were less numerous in *C*. *felis*, correlating with reduced incidence of partially digested blood material in the cat flea foregut. Thus, the efficient EPT vectors *O*. *montana* and *X*. *cheopis* regularly develop PIER, but the poor EPT vector *C*. *felis* does not.

### PIER is associated with obstruction of the proventriculus within 72 hours

**Fig 5:**
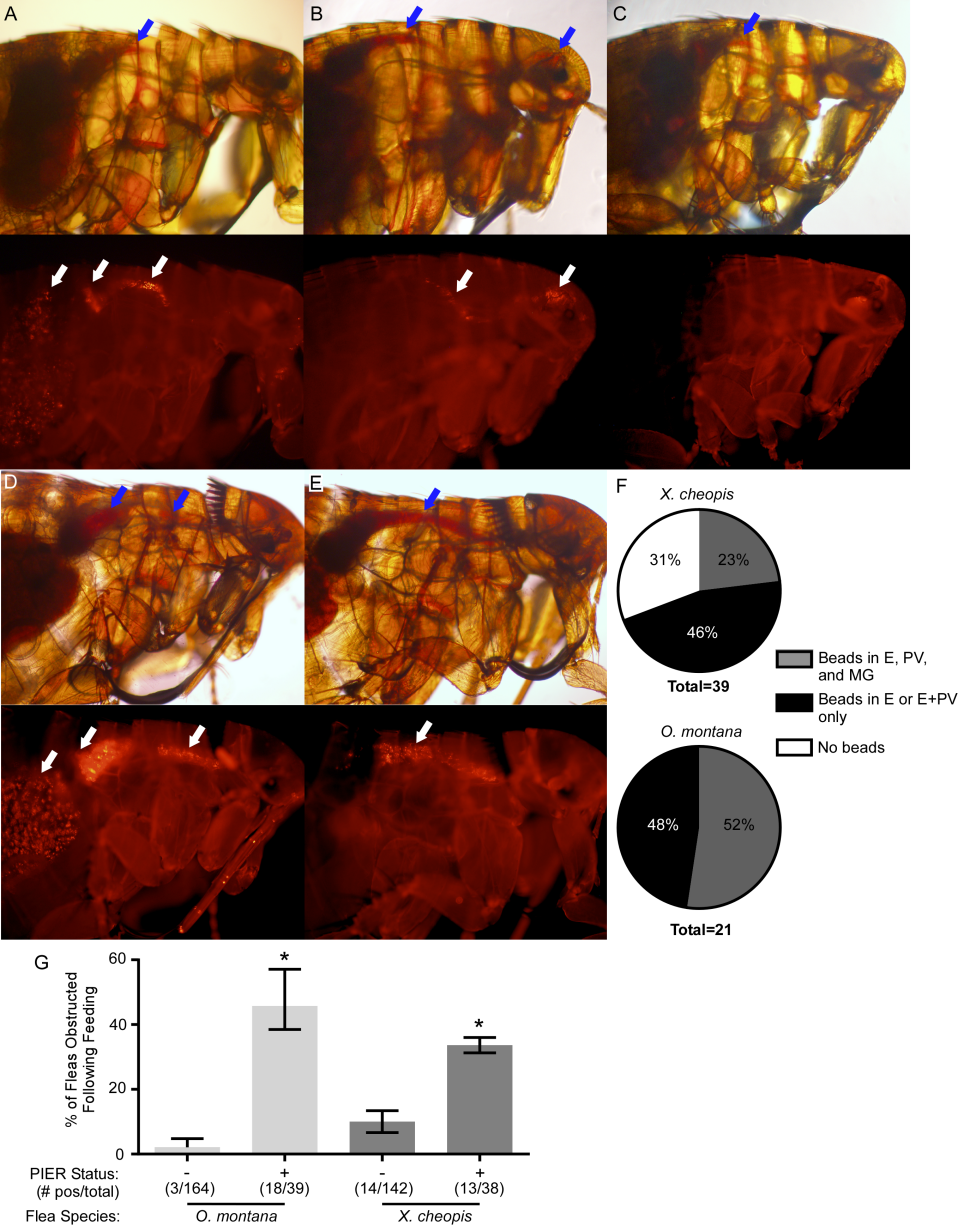
PIER correlates with increased likelihood of proventricular obstruction 3 days after infection. *X*. *cheopis* or *O*. *montana* were infected using rat blood containing ~1 x 10^9^ CFU/ml KIM6+ (pAcGFP1) *Y*. *pestis*. 3 days after infection, fleas were separated into PIER^+^ and PIER^-^ groups, fed on sterile rat blood containing fluorescent beads, screened for signs of digestive tract obstruction, and imaged to determine localization of beads in the digestive tract. Red blood (blue arrows) and fluorescent beads (white arrows) in the esophagus are evidence of proventricular obstruction. Most fleas initially diagnosed with obstruction (red blood in the esophagus) could be verified as being either partially blocked (beads present in the esophagus, proventriculus, and midgut, (A and D) or fully blocked (beads present only in the esophagus and/or proventriculus, (B and E). For *X*. *cheopis*, but not *O*. *montana*, a subset (31%) of the fleas initially diagnosed as obstructed based on visualizing red blood in the esophagus were false-positives- these fleas had not actually fed because no beads were present anywhere in the digestive tract (C). (F) Cumulative distribution of partially blocked, fully blocked, and misdiagnosed (false-positive) *X*. *cheopis* and *O*. *montana* fleas. (G) Percentages of PIER^+^ and PIER^-^ *X*. *cheopis* and *O*. *montana* with proventricular obstruction 3 days after infection, as diagnosed after feeding on fluorescent bead-laden blood. *p < 0.001 compared to PIER^-^ conspecifics by chi-square test. The cumulative mean and range of 2–3 independent experiments is shown.

To discern whether PIER interfered with ingestion of blood, *X*. *cheopis* and *O*. *montana* infected using rat blood were subsequently fed a sterile blood meal containing fluorescent beads 3 days after the initial infection. Prior to being fed on the bead-laden blood, fleas with PIER were separated from those without PIER. Following the blood meal, fleas that appeared to have proventricular obstruction (presence of fresh blood in the esophagus) were imaged to track the location of fluorescent beads in the digestive tract. Most fleas diagnosed with obstruction appeared to be either partially blocked (23–52%; beads present in both the esophagus and the midgut) (Fig 5A and 5D) or fully blocked (46–48%; beads present in the esophagus and proventriculus only) (Fig 5B and 5E). However, 31% of *X*. *cheopis* diagnosed with proventricular obstruction were false positives, with no evidence of beads in the digestive tract (Fig 5C and 5F). These fleas had not actually fed; the initial diagnosis of obstruction was due to PIER-derived blood that remained red for at least 3 days post-infection. This was not observed for *O*. *montana*, in which PIER-derived blood in the esophagus was dark brown and easily distinguished from recently ingested blood, and those diagnosed with obstruction always had beads present in the foregut.

*O*. *montana* or *X*. *cheopis* diagnosed with PIER prior to feeding on the bead-laden blood were 3 to 25 times more likely to have evidence of at least partial obstruction of the proventriculus (fresh blood and beads trapped in the esophagus) compared to those without PIER (Fig 5G). Fresh red blood trapped in the esophagus after feeding is typically observed in fleas that transmit by the biofilm-dependent regurgitative mechanism [2, 8]. These data indicate that PIER often causes sufficient proventricular obstruction to interfere with blood feeding.

### Early-phase transmission efficiency correlates with the source of host blood, PIER, and digestive tract obstruction

**Fig 6:**
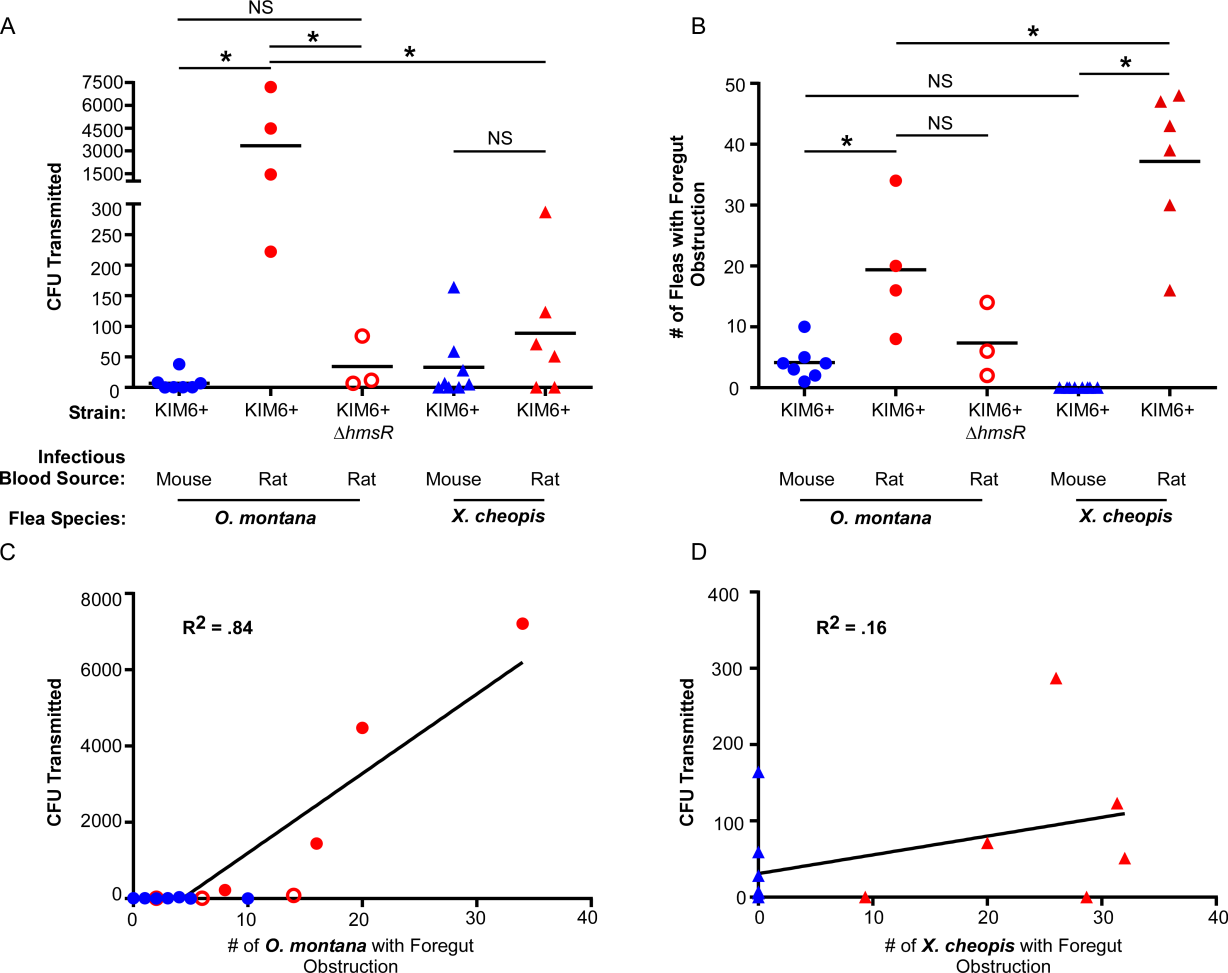
Biofilm-dependent transmission can augment early-phase transmission, but is dependent on flea species and infectious blood meal source. (A) *Y*. *pestis* CFU transmitted in *in vitro* mass transmission experiments by groups of *O*. *montana* or *X*. *cheopis* 3 days after infection with *Y*. *pestis* KIM6+ (pAcGFP1) or KIM6+ Δ*hmsR* pAcGFP1) in mouse (blue symbols) or rat blood (red symbols). (B) Number of infected fleas from each transmission experiment that were diagnosed as either partially or completely blocked after feeding (fresh red blood present in both the esophagus and midgut or in the esophagus only). (C) Linear regression of the number of infected fleas with foregut obstruction (partial or complete blockage) and CFU recovered following transmission assays for *O*. *montana* (p < 0.0001) and (D) *X*. *cheopis* (p = 0.15). The coefficient of determination (R^2^) is listed on the graph. Each symbol on all four graphs represents data from a single independent transmission test (see Table 2 for details). Horizontal bars (A and B) indicate the mean; *p < 0.01 (A) or *p < 0.05 (B) by one-way ANOVA with Tukey’s post-test. NS = not significant. CFU data for mouse blood experiments in (A) are previously published [6].

To test whether the source of the infectious blood meal affected transmission of *Y*. *pestis*, groups of ~200 *O*. *montana* or *X*. *cheopis* fleas, infected 3 days earlier with mouse or rat blood containing high concentrations of *Y*. *pestis* (>1 x 10^8^ CFU/ml; Table 2), were allowed to feed on sterile blood. Immediately following the 1.5 h feeding period, the number of CFU transmitted was determined by plate count. In addition, fleas were screened for evidence of feeding and for foregut obstruction. *O*. *montana* and *X*. *cheopis* infected using rat blood transmitted ~420- and 3-fold more CFU, respectively, than those infected using mouse blood (Fig 6A, Table 2). Significantly more fleas infected using rat blood had foregut obstruction compared to those infected using mouse blood (Fig 6B). Surprisingly, the increase in CFU transmitted by *O*. *montana* infected with rat blood was biofilm-dependent; fleas infected with *Y*. *pestis* KIM6+ Δ*hmsR*, a strain unable to produce and export the exopolysaccharide needed for mature biofilm formation, transmitted 98-fold fewer CFUs than *O*. *montana* infected with the parental Hms+ strain. The number of CFU transmitted and the number of fleas with foregut obstruction correlated well for infected *O*. *montana* (R^2^ = 0.84), but not for *X*. *cheopis* (R^2^ = 0.16) (Fig 6C and 6D). False-positive misdiagnosis of a portion of the obstructed *X*. *cheopis* may partially explain the poor correlation between foregut obstruction and CFU transmitted in EPT assays (Fig 5F). The data suggest that fleas infected using rat blood develop proventricular obstruction at a faster rate, reducing the extrinsic incubation period required for BDT. Thus, in certain conditions BDT can play a role as soon as 3 days after infection, overlapping with the traditional EPT timeframe (1–7 days post-infection) [39].

**Table 2:** Summary of early-phase (day 3 post-infection) in vitro mass transmission experiments.

	*Y*. *pestis* CFU/ml in infectious blood meal	Total # of Fleas	# of Fleas That Fed (%)	# of Obstructed Fleas	CFU Transmitted	Average CFU Transmitted per Flea	Post-Feed Flea Infection Rate and Mean Bacterial Load†
***O*. *montana* infected with *Y*. *pestis* KIM6+ in mouse blood**^**§**^							
Expt 1	1.5x10^9^	212	124 (58.5%)	4	38	0.84	36% (5.1x10^4^)
Expt 2	2.8x10^8^	147	132 (89.8%)	5	0	0	10% (1.4x10^5^)
Expt 3	4x10^8^	213	136 (63.9%)	4	0	0	35% (2.1x10^5^)
Expt 4	1.9x10^8^	210	173 (82.4%)	2	1	0.01	65% (3.1x10^5^)
Expt 5	1.1x10^9^	270	132 (48.9%)	10	0	0	ND
Expt 6	1.9x10^9^	187	**1**44 (77.0%)	1	8	0.22	25% (1.1x10^5^)
Expt 7	1.4x10^9^	205	202 (98.5%)	3	7	0.09	40% (1.8x10^5^)
**Average**	**9.7x10**^**8**^	**206**	**149 (72%)**	**4**	**8**	**0.17**	**37.6% (1.7x10**^**5**^**)**
***O*. *montana* infected with *Y*. *pestis* KIM6+ in rat blood**							
Expt 1	1.1x10^9^	188	160 (85.1%)	20	4476	27.98	100% (1.3x10^6^)
Expt 2	8.5x10^8^	183	157 (85.8%)	16	1440	9.17	100% (1.2x10^6^)
Expt 3	1.1x10^9^	201	199 (99.0%)	8	222	1.31	85% (1.5x10^6^)
Expt 4	5.5x10^8^	198	173 (87.4%)	34	7210	41.68	100% (4.3x10^6^)
**Average**	**8.9x10**^**8**^	**195**	**172 (88%)**	**20**	**3337**	20.03*	96.3%** (2.1x10^6^)
***O*. *montana* infected with *Y*. *pestis* KIM6+ (Δ*hmsR*) in rat blood**							
Expt 1	1.8x10^9^	203	180 (88.7%)	14	84	0.85	55% (7.3x10^5^)
Expt 2	7.0x10^8^	189	143 (75.7%)	6	12	0.17	50% (2.9x10^5^)
Expt 3	8.0x10^8^	190	163 (85.8%)	2	7	0.17	25% (3.6x10^5^)
**Average**	**1.1x10**^**9**^	**194**	**162 (84%)**	**7**	**34**	**0.4**	**43.3% (4.6x10**^**5**^**)**
***X*. *cheopis* infected with *Y*. *pestis* KIM6+ in mouse blood**^**§**^							
Expt 1	1.1x10^9^	456	139 (30.5%)	0	59	0.42	ND
Expt 2	1.0x10^9^	199	103 (51.8%)	0	0	0	100% (1.2x10^6^)
Expt 3	9.0x10^8^	205	156 (76.1%)	0	5	0.03	95% (1.3x10^6^)
Expt 4	4.9x10^8^	180	148 (82.2%)	0	164	1.23	90% (5.5x10^5^)
Expt 5	3.6x10^8^	204	153 (75.0%)	0	7	0.05	95% (1.2x10^6^)
Expt 6	2.0x10^9^	198	145 (73.2%)	0	0	0.00	85% (5x10^5^)
Expt 7	2.5x10^8^	202	148 (73.3%)	0	0	0.00	100% (7.3x10^5^)
Expt 8	3.2x10^8^	201	113 (56.2%)	0	28	0.26	95% (7.9x10^5^)
**Average**	**8.0x10**^**8**^	**231**	**138 (60%)**	**0**	**33**	**0.22**	94.3%** (9x10^5^)
***X*. *cheopis* infected with *Y*. *pestis* KIM6+ in rat blood**							
Expt 1	1.1x10^9^	203	152 (74.9%)	48	51	0.45	75% (8.8x10^5^)
Expt 2	1.1x10^9^	203	107 (52.7%)	14	0	0	80% (7.8x10^5^)
Expt 3	2x10^9^	199	135 (67.8%)	30	71	0.53	100% (1.7x10^6^)
Expt 4	9.7x10^8^	210	129 (61.4%)	39	287	2.78	80% (1.3x10^6^)
Expt 5	8.8x10^8^	205	114 (55.6%)	47	123	1.14	95% (7.3x10^5^)
Expt 6	7x10^8^	209	135 (64.6%)	43	0	0	75% (5.7x10^5^)
**Average**	**1.1x10**^**9**^	**205**	**129 (63%)**	**37**	**89**	**0.82**	84.2%** (1x10^6^)

ND = Not Determined

†All fleas sampled were infected on Day 0, n = 10–20

^§^Data from reference [6]

* p< 0.05 by Student’s t-test compared to *X*. *cheopis* rat blood group

**p< 0.0001 by chi-square test compared to *O*. *montana* mouse blood or rat blood (Δ*hmsR*) groups

As previously reported, flea infection rates following the transmission tests (3 day post-infection maintenance feed) were significantly lower in *O*. *montana* that had been infected using mouse blood rather than rat blood (38% vs. 96%, Table 2) [18]. Infection rates of fleas in the mouse blood group were comparable to those of fleas that had been infected with KIM6+ Δ*hmsR* in rat blood (43%), a strain that cannot permanently colonize the proventriculus [8, 40]. In addition, bacterial burdens of *O*. *montana* from the mouse blood group that remained infected were about 10-fold lower than the rat blood group (Table 2).

We considered the possibility that differences in feeding rate may explain why *O*. *montana* transmitted more CFU than *X*. *cheopis* infected using rat blood. On average, 129–138 (60–63%) infected *X*. *cheopis* fed during the EPT transmission assays, whereas 149–172 (72–88%) infected *O*. *montana* fed (Table 2). However, even when estimating CFU transmitted on a per flea basis under optimal conditions (rat blood infection), infected *O*. *montana* transmitted significantly more *Y*. *pestis* than *X*. *cheopis* (20 vs 0.8, respectively; Table 2).

## Discussion

Between 1905–1916, soon after fleas were proven to be the important vectors of plague, two temporally distinct phases of flea-borne transmission were described [3]. What became known as mass transmission, and more recently termed early-phase transmission (EPT), occurs when groups of fleas feed on a naïve animal during the first week after their infectious blood meal, and wanes thereafter. A later phase of transmission occurs after *Y*. *pestis* produces sufficient stable biofilm growth in the proventriculus to partially or completely block normal blood flow during feeding, and is routinely effected by a single flea during continuous feeding attempts as it gradually starves.

Recent work has shed new light on both modes of transmission. EPT of *Y*. *pestis* was long assumed to be an example of mechanical transmission, and EPT of *Rickettsia felis* by cat fleas occurs by this mechanism [41]. However, the fact that different fleas vary greatly in their EPT efficiency following infection with *Y*. *pestis*, and that early-phase mass transmission by fleas infected with *Y*. *pseudotuberculosis* is never detected, argue against a simple mechanical mechanism, because all fleas would have been expected to soil their mouthparts and transmit equivalently by this means [42, 43]. We recently proposed that EPT, like BDT, occurs via regurgitation of bacteria from the proventriculus [8]. This was based on the striking observation that the proventriculus is a primary site of infection that is often heavily colonized within a day after an infectious blood meal. This was unexpected, because the long-standing conception has been that the midgut is infected first and that the proventriculus is not colonized until a few days later in *X*. *cheopis*, and later and less often in other fleas [12, 14]. However, we found that in the majority of all three flea species examined (*X*. *cheopis*, *O*. *montana*, and *C*. *felis*), large aggregates of bacteria, surrounded by an amorphous, brown-colored matrix derived from digestive products of the blood meal, were present in both the proventriculus and the midgut 1–3 days after feeding on blood containing the high concentrations of *Y*. *pestis* required to sustain EPT [8].

**Fig 7:**
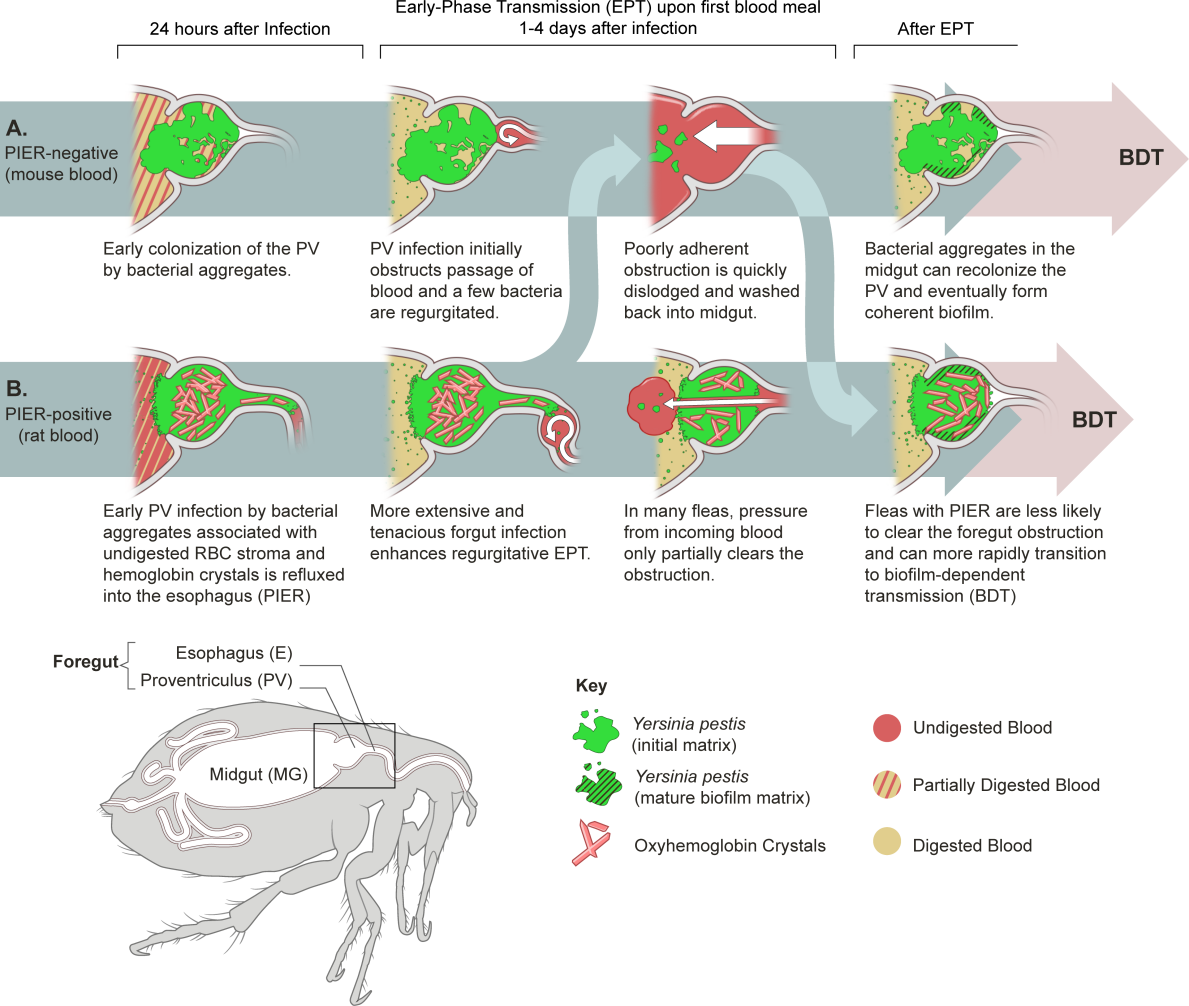
Model of early-phase regurgitative transmission of *Y*. *pestis* by PIER positive and PIER negative fleas. Following an infectious blood meal, fleas rapidly lyse red blood cells (RBC) and hemoglobin is released. Within a few hours, *Y*. *pestis* coalesces into aggregates that are enveloped in an exogenous matrix that is derived from blood digestion products [8]. Depending on the blood source, incompletely digested RBCs and oxyhemoglobin crystals can associate with the bacterial aggregates in the midgut. Normal peristalsis and proventricular pulsations, which continue long after feeding, draw the aggregates into the proventriculus, and some of them lodge there. According to the model, when fleas with extensive proventricular colonization feed again 1–3 days after infection, the incoming blood flow is sufficiently impeded to result in regurgitation of bacteria. Early proventricular colonization and early-phase transmission do not require mature biofilm formation and the permanent proventricular colonization that is mediated by the *Y*. *pestis hmsHFRS* genes [8, 15]. (A) In PIER-negative fleas, RBCs are digested quickly and hemoglobin remains soluble. The bacterial aggregates in the proventriculus of heavily colonized fleas withstand the initial pulses of incoming blood, which can result in regurgitative transmission of a few bacteria. However, the obstruction is ephemeral and incoming blood quickly dislodges most or all of the poorly adherent bacterial aggregates back into the midgut. The proventriculus can later be recolonized permanently by an Hms-dependent biofilm. (B) In PIER-positive fleas, less rapid RBC digestion and formation of oxyhemoglobin crystals correlate with a more tenacious, expansive proventricular colonization and extrusion of infected blood into the esophagus (post-infection esophageal reflux; PIER). The obstruction in the PV is more resistant to dislodgement and bacteria are already present in the esophagus, both of which enhance regurgitative EPT. Incoming blood ultimately channels through the proventricular obstruction of most fleas and enters the midgut. However, because PIER tends to stabilize the initial proventricular colonization, the extrinsic incubation period for biofilm-dependent transmission (BDT) may be shortened, and the two phases of transmission may temporally overlap in populations of infected fleas. Figure adapted from reference [8].

The results presented here support the regurgitative mechanism model for EPT and provide new details. According to our model ([8]; Fig 7), those fleas with an initial heavy proventricular colonization are competent early-phase vectors, because blood flow through the proventriculus is sufficiently restricted to result in reflux of blood mixed with bacteria back into the bite site. Here we show that the makeup of the proventricular bacterial aggregates differs with different infectious blood sources, and that this can lead to an extension of initial proventricular infection forward into the esophagus and affect EPT efficiency.

Within 24 h after ingesting bacteremic rat or guinea pig blood, *X*. *cheopis* and *O*. *montana* fleas often regurgitated some blood meal material containing *Y*. *pestis* into the esophagus (post-infection esophageal reflux; PIER). Although primary proventricular colonization occurs in 67–100% of *X*. *cheopis* and *O*. *montana* fleas after they feed on bacteremic mouse blood [8], they rarely exhibit PIER. PIER-inducing blood sources affected early proventricular colonization in two ways. In addition to the presence of infected blood meal regurgitant in the esophagus, fleas with PIER exhibited a more prominent and tenacious early colonization of the proventriculus. This was evidenced by greater expansion of the valve (Fig 2A) and an increased incidence of obstruction of blood flow into the midgut upon the first maintenance feed 3 days after infection compared to fleas without PIER (Fig 5; Table 2). The extrusion of bacteria forward into the esophagus and the enhanced proventricular obstruction associated with PIER would both be predicted to enhance a regurgitative EPT mechanism. EPT efficiency, as assessed by the average CFU transmitted per flea, was in fact ~4-fold and ~100-fold higher for PIER^+^ than for PIER^-^ *X*. *cheopis* and *O*. *montana*, respectively (Table 2, Fig 6). Interestingly, most of this increase observed in *O*. *montana* was dependent on an intact *hmsHFRS* operon, which is required for *Y*. *pestis* to produce the poly-ß-1,6-*N*-acetyl-D-glucosamine exopolysaccharide of a mature biofilm and for BDT [5]. This result indicates that, although PIER fortifies initial proventricular colonization and obstruction, even with PIER the early-phase foregut obstruction generates less regurgitative pressure than during later BDT. The CFU transmitted per flea by the later, biofilm-dependent mechanism can be orders of magnitude higher than by EPT [6, 42, 44].

Evidence that EPT is due to regurgitation from the flea foregut includes: 1) The proventriculus (or proventriculus and esophagus in the case of PIER) is often heavily colonized by *Y*. *pestis* within 24 h after a highly bacteremic blood meal in all flea species examined ([8]; Fig 2). 2) The complex aggregate of bacteria and digestive byproducts that accumulates in the foregut frequently obstructs ingestion during the next blood meal, and this correlates with increased EPT efficiency (Fig 6; Table 2). 3) Flea species whose behavior and physiology promotes retention of bacteria in the foregut and induction of PIER (*O*. *montana* and *X*. *cheopis*) are relatively efficient EPT vectors whereas those that do not (*C*. *felis*) are inefficient (Fig 4) [6, 43, 45]. 4) *Y*. *pestis* Hms mutants unable to synthesize and export the exopolysaccharide required for mature biofilm growth nevertheless form typical aggregates, transiently colonize the proventriculus, and are transmitted during the early-phase window (Fig 6 and Table 2) [8, 15]. 5) EPT efficiency is not affected by loss of the phospholipase D activity encoded by the *Y*. *pestis ymt* gene, which enhances bacterial survival in the midgut but is not required for infection of the proventriculus [40, 46].

PIER correlated strongly with the use of rodent blood sources that are characterized by poorly soluble hemoglobin and a relatively slow digestion rate in the flea. The major constituent of a blood meal is water, much of which is rapidly eliminated through diuresis, concentrating the formed elements of the blood into a bolus [21, 36]. Hemolysis with release of hemoglobin occurs within 3–6 h after a blood meal [21, 47]. Free oxyhemoglobin can crystalize in the digestive tract, depending on the solubility characteristics of the particular species of hemoglobin [36]. The rate that oxyhemoglobin crystals and RBC stroma are processed and eliminated varies in different flea species and for different blood types (Fig 2 and Fig 4). PIER-inducing rat and guinea pig blood is digested more slowly in the midgut and the hemoglobin of these two species is poorly soluble and crystalizes rapidly, whereas non-PIER-inducing mouse blood is typically digested within 12–24 h and its hemoglobin does not readily form crystals [31, 32, 36]. Notably, *X*. *cheopis* that feed on sterile rat blood digest their entire blood meal and the hemoglobin crystals that form disappear within 20–30 h of feeding [36]. This timeframe of RBC hemolysis and subsequent hemoglobin crystal formation roughly correlates with the earliest observations of PIER in infected fleas (8 h post-infection). Interestingly, fleas that consumed sterile blood of any type or those that ingested *E*. *coli* or heat-killed *Y*. *pestis* rarely developed PIER (Fig 1A and 1C), indicating that metabolic activity of *Y*. *pestis* is important for PIER induction.

We hypothesize that PIER occurs when accumulation of partially digested blood meal material and oxyhemoglobin crystal prevents the proventricular spines from overlapping and sealing off the midgut, and bacteria and blood debris are pushed into the esophagus by proventricular contractions and midgut peristalsis (Fig 7B). Typically, the backward directed proventricular spines that circle the lumen of the proventriculus are thought to partially or completely interlock, preventing backflow of blood into the esophagus [7]. The flea midgut and proventriculus musculature continues to contract long after a blood meal, churning and passaging the gut contents between the two compartments [48]. The proventriculus of fleas that develop PIER appears to be enlarged (Figs 1B and 5A–5E) because it is packed with bacteria, partially digested blood, and hemoglobin crystals that spread the proventricular spines farther apart (Figs 2A, 2C and 4B). Consistent with our hypothesis, PIER appeared to inhibit normal post-feeding proventricular contractions to some degree, although midgut peristalsis still occurred (S1 and S2 Videos). Taken together, our results indicate that metabolic activity of *Y*. *pestis*, in addition to bacterial aggregation and colonization of the proventriculus, is important for PIER induction.

We considered the possibility that differential agglutination of mouse and rat RBCs by *Y*. *pestis* could partially account for the differences in proventricular colonization and induction of PIER because *Yersinia* pH 6 antigen (Psa) is expressed at 37°C (the growth temperature of the bacteria added to the infectious blood meals) and is a known hemagluttinin [49]. However, comparative hemagglutination assays revealed that RBCs from mice and rats were agglutinated equivalently by KIM6+ (S3 Fig). It is also important to note that while fibrin clots were once hypothesized to be important for chronic infection of the flea proventriculus, this has been ruled out [50, 51]. As such, we used either defibrinated or heparinized blood from mice or rats for our *in vitro* flea infections and showed that the incidence rates of PIER were equivalent for both methods of anti-coagulation (Fig 1C and S1 Fig). The results indicate that addition of sodium heparin to infectious rodent blood does not affect PIER for rodent fleas. The fact that rat RBCs alone are sufficient for PIER induction (S1 Fig) also indicates that plasma components are not required.

Some differences were observed in the effect that blood source had on early infection characteristics in the three flea species examined. Unlike the two rodent flea species, PIER did not develop in cat fleas infected using rat blood. This may be because cat fleas process their blood meals more quickly, making it less likely that hemoglobin crystals and RBC stroma accumulate (Fig 4E). In addition, cat fleas have a thick band of musculature that narrows and constricts the esophagus at its intersection with the proventriculus, which may act as a sphincter that prevents backflow of material into the esophagus [43]. This musculature is diminished or absent in *X*. *cheopis* and *O*. *montana* [8, 43]. We hypothesize that these anatomical and physiological traits make it more difficult for *Y*. *pestis* to persistently colonize the cat flea proventriculus, less likely to develop PIER, and less efficient at regurgitative transmission, both in the early phase and later [43].

Differences were also observed between *X*. *cheopis* and *O*. *montana*. Consistent with a previous study [18], the infection rate and bacterial burden for *O*. *montana* at 3 days post-infection was significantly lower when the infectious blood meal was composed of mouse blood compared to rat blood (Table 2). In contrast, *X*. *cheopis* infection rates and bacterial loads were equivalent with the two blood sources. The rates were also lower for *O*. *montana* infected with the *Y*. *pestis* KIM6+ Δ*hmsR* mutant than with KIM6+ wild-type in rat blood.

A second difference between *O*. *montana* and *X*. *cheopis* was the much greater effect that rat blood had on the number of bacteria transmitted 3 days post-infection (Table 2; Fig 6A). This increased transmission efficiency was biofilm-dependent as well as blood-source-dependent, because it was not seen with the *hmsR* mutant. This indicates that, in some fleas, sufficient HmsHFRS-dependent exopolysaccharide is produced to stabilize the proventricular aggregate, thereby increasing regurgitative transmission efficiency. We have recently shown that ~1–3% of *O*. *montana* infected using mouse blood become partially blocked within 3 days of infection and ~25% are fully blocked by 6 days post-infection [6]. The great gerbil flea *Xenopsylla skrjabini* can also become fully blocked by 3 days after infection [52]. In the present study, 4–20% of *O*. *montana* infected using rat blood appear to become either partially or fully blocked within 3 days of infection (Table 2; Fig 5), generally sooner and with greater frequency than those infected with mouse blood (Fig 6B). Furthermore, biofilm-dependent transmission is not dependent on complete blockage of the foregut; partially blocked fleas can also transmit [7, 8]. Thus, although the extrinsic incubation period required for biofilm-dependent transmission is generally reported as 1–2 weeks after infection for *X*. *cheopis*, it can be as short as a few days, suggesting that early-phase and biofilm-dependent transmission do not always occur in two distinct temporal phases.

Infections using rat blood also increased the number of *Y*. *pestis* transmitted by *X*. *cheopis* during the early-phase, but not to the extent seen for *O*. *montana* (Table 2, Fig 6A). This was surprising, because more *X*. *cheopis* than *O*. *montana* infected using rat blood were diagnosed with foregut obstruction (partial or full blockage) following the day-3 EPT feeds. Given this, it might be expected that groups of *X*. *cheopis* would transmit more CFU than *O*. *montana*. One confounding variable was that up to ~30% of infected *X*. *cheopis* identified as being obstructed in our EPT experiments were likely false positives (Figs 5B, 6C and 6F). The portion of the blood meal refluxed into the esophagus (by PIER) appears to be digested more slowly than the contents of the midgut, causing it to remain bright red for some infected *X*. *cheopis*. This limited our ability to visually diagnose obstruction in *X*. *cheopis* infected using rat blood when fed within 3 days of the infectious blood meal. Additionally, we have shown that *O*. *montana* transmits more *Y*. *pestis* than *X*. *cheopis* by the BDT mechanism, despite the fact that *X*. *cheopis* has a higher blockage rate [6]. We have hypothesized that these differences in transmission efficiency may be related to anatomical features of the flea foregut; specifically, the *O*. *montana* foregut appears more susceptible to biofilm-induced distension at or near the esophageal-proventricular junction [6]. This may affect the hydrodynamics of transmission, in that a greater surface area of the infectious biofilm is exposed to incoming blood, increasing the likelihood that bacteria are dissociated and transmitted. Collectively, the data lead us to conclude that *O*. *montana* is a more efficient vector since it becomes blocked more quickly than *X*. *cheopis* and transmits greater numbers of *Y*. *pestis* CFU (Fig 6) [6]. Our data indicate that rat blood-based infections decrease the time required for *O*. *montana* to transition from EPT to BDT, resulting in temporal overlap of the two modes of transmission. Based on this study and recent findings indicating that *O*. *montana* readily become completely blocked shortly after infection (mean = 9.6 days), even when mouse blood is used, and survive for extended periods after becoming blocked (mean = 7 days, range = 1–16 days), we think it likely that both EPT and BDT are simultaneously operative during epizootics involving the ground squirrel hosts of *O*. *montana* [6]. The high vector competence of *O*. *montana* may in part account for the rapidity with which *Y*. *pestis* spread throughout the western United States after it was introduced shortly before the turn of the 20^th^ century, particularly if ground squirrel blood is PIER-inducing.

A previous study that measured EPT efficiency by groups of ~10 infected *O*. *montana* using rat blood found that biofilm-deficient *Y*. *pestis* Δ*hmsR* or Δ*hmsT* strains were transmitted at least as well as the parental strain [15]. In partial agreement with these observations, biofilm production was not requisite for EPT in our mass transmission experiments; however, significantly more CFU of the parental Hms+ *Y*. *pestis* than the Δ*hmsR* mutant were transmitted (Fig 6A and Table 2), which would be predicted to increase disease incidence following EPT challenge. The two studies used dissimilar experimental models, however: small groups of infected fleas used to challenge Swiss-Webster mice, with disease or seroconversion as the readout; versus large groups of infected fleas feeding on sterile blood in an artificial feeding device, with the number of CFU recovered from the blood reservoir being the readout. Swiss-Webster mice are highly susceptible to plague by peripheral routes (<10 CFU), making that model of EPT highly sensitive for detecting transmission of few CFU [22, 53]. Mice which seroconverted but did not develop fulminant disease were included in calculations of transmission efficiency, which indicates that the number of CFU transmitted was sometimes below the LD50 [15]. An advantage of the artificial feeding model of transmission is that it allows for quantification of the total CFU transmitted by groups of fleas. We hypothesize that the large increase in CFU transmitted by *O*. *montana* infected with the Hms^+^ compared to the Hms^-^ *Y*. *pestis* in our mass transmission assays came from a small number of the 157–199 fleas in which enough of the Hms-dependent extracellular matrix had been produced to effect BDT (Table 2). Flea transmission efficiency, in general, is low by both EPT and BDT mechanisms; however individual blocked fleas can transmit >1,000 CFU by BDT, much higher than is transmitted by EPT [44]. Our use of large pools of fleas increases the likelihood of detecting these relatively infrequent early BDT events.

Hemoglobin solubility is highly variable among the Muridae (mice, rats, and gerbils) [31]. Digestion of rat blood resulted in the formation of abundant oxyhemoglobin crystals in the flea digestive tract, but digestion of mouse or gerbil blood did not (Fig 3). Interestingly, the hemoglobin of the Sciuridae family of rodents, which includes squirrels, marmots, and prairie dogs that are susceptible to plague epizootics, has been described as poorly soluble and prone to crystal formation, suggesting that fleas that feed on ground squirrels or prairie dogs would develop PIER after feeding on highly bacteremic blood [31]. Our findings provide a possible explanation as to why the Sciuridae and their fleas are prominent in plague ecology and epizootics spread rapidly in their populations: fleas that ingest bacteremic blood with poorly soluble hemoglobin can rapidly develop a foregut obstruction that is resistant to dislodgement and promotes regurgitative transmission of *Y*. *pestis*. Future studies of vector competence using rodent blood obtained from other hosts susceptible to *Y*. *pestis* such as great gerbils, prairie dogs, and ground squirrels will provide valuable information for generating hypotheses about *Y*. *pestis* transmission dynamics during plague outbreaks.

## Materials and methods

### Flea infection

*Yersinia pestis* KIM strains and *Escherichia coli* cloning strains were grown at 37°C in brain-heart infusion (BHI) broth supplemented with hemin (10 μg/ml) as described previously [43] (Table 1). Unless otherwise noted, KIM strains contained pAcGFP1 (Clontech; Mountain View, CA). *E*. *coli* DH5α was transformed with pCH16, a plasmid that encodes Yersinia murine toxin (Ymt), a phospholipase D enzyme needed for bacterial colonization of the flea midgut [28] (Table 1). *C*. *felis*, *O*. *montana*, and *X*. *cheopis* fleas from colonies established at Rocky Mountain Laboratories (RML) were starved for 4–5 days prior to infection. Groups of about 150–300 fleas were allowed to feed from an artificial feeding device containing 5–6 ml of sterile blood or blood that contained 1 x 10^8^–1 x 10^9^ bacterial CFU/ml [54]. For other infection experiments, the bacteria were added to 5 ml of washed rat RBCs in PBS or to 5 ml of defibrinated mouse blood to which the RBC stroma and oxyhemoglobin crystals derived from 5 ml of rat blood (prepared as described below) had been added. For experiments using heat-killed bacteria, bacteria were concentrated, suspended in 1 ml of sterile phosphate-buffered saline (PBS), and incubated at 65°C for 20 minutes prior to addition to the blood meal. Loss of viability was confirmed by plating 50 μl of the heat-killed bacterial suspension on blood agar. Blood sources for the infections were defibrinated or heparinized Sprague-Dawley rat (*Rattus norvegicus*), defibrinated or heparinized Swiss-Webster mouse (*Mus musculus*), defibrinated Hartley guinea pig (*Cavia porcellus*), or defibrinated Mongolian gerbil (*Meriones unguiculatus*). Most blood was purchased from Bioreclamation IVT (New York). Sodium heparin treated mouse blood was collected on site. After a 1.5 h (*O*. *montana* and *X*. *cheopis*) or 4 h (*C*. *felis*) feeding period, fleas were screened for evidence of feeding and only those that had taken an infectious blood meal were used in experiments. When groups of fleas were not being screened for PIER or used in EPT assays, they were housed in plastic capsules and kept at 21°C, 75% relative humidity [54].

### Bacterial mutagenesis

The *Y*. *pestis hmsR* mutant, which lacks 681 base pairs of the open reading frame (amino acids 63–290) including the entirety of the glycosyltransferase domain, was generated by allelic exchange using the pCVD442*hmsR* suicide vector (Table 1). An in-frame deletion (described above) was generated by inverse PCR of the pDONR221*hmsR* Gateway vector (Pathogen Functional Genomics Resources Center, J. Craig Venter Institute), which contains the *Y*. *pestis* KIM *hmsR* ORF as well as ~500 base pairs up- and downstream, using 5’ phosphorylated primers GGAAACAGCGTCTCCGCTGGGCGCAAGGCGGTGCGG and CCCACGGCCAGTGCCTCTCTCGGCGTAGCCAGAAATAGC. The deletion fragment was amplified from the cloning vector by PCR using primers CGCTCTAGAAGCGGTGGACTCGTTACAAG and GCGGAGCTCACTGGAGCAACTTCTGGCAG and was ligated into pCVD442 using restriction sites XbaI and SacI. Cointegrants were generated in *Y*. *pestis* KIM6+ by conjugation with *E*. *coli* S17-1 (pCVD442*hmsR*). Following sucrose selection, the *hmsR* mutant was validated by plating on Congo red agar and by PCR.

### Blood fractionation, oxyhemoglobin crystal analysis, and hemagglutination assay

Constituents of whole blood were separated by centrifugation at 1500–2000 x g, plasma was removed, and RBCs were washed 3 times with sterile PBS. For hemolysis, RBCs were resuspended in dH_2_O equal to the volume of packed RBCs for 10 minutes at room temperature or were frozen and thawed (20 minutes, -80°C). Following hemolysis, RBC stroma and oxyhemoglobin crystals were isolated by centrifugation at 10,000 rpm for 10 minutes. Absorbance of oxyhemoglobin crystals was measured using a TECAN Safire^2^ plate reader.

For hemagglutination assays, *Y*. *pestis* was cultured at 37°C in BHI, resuspended in sterile PBS to a concentration of ~5x10^7^ CFU/ml; and 2-fold serial dilutions were made in a 96-well round-bottomed polystyrene dish. Washed mouse or rat RBCs were added to each well to a final concentration of 1% and thoroughly mixed with bacteria. Wells that contained unsedimented RBCs after 3h at room temperature (persistence of a hazy suspension) were scored as positive for agglutination.

### Monitoring flea PIER and proventricular obstruction

24 h following infection, fleas were immobilized by placing them on ice and screened using a dissecting microscope for signs of post-infection esophageal reflux (PIER). PIER was defined as the presence of red blood in the esophagus noticeably beyond the esophageal-proventricular junction following an infectious blood meal but prior to a subsequent maintenance feed on sterile blood. The number of fleas positive for PIER was recorded and fleas were returned to the capsule. For fluorescent tracking of PIER, 1 μm red fluorescent (580/605 nm) sulfate FluoSpheres (Thermo Fisher Scientific; Waltham, MA) was added to the infectious blood meal to a final concentration of ~3.6 x 10^8^ beads/ml. To evaluate and verify proventricular obstruction, fleas were fed on sterile rat blood containing fluorescent beads 3 days after being infected using rat blood without beads.

### Flea infection rate and CFU counts

Samples of 10–20 female fleas were collected immediately after their infectious blood meal and at 24 or 72 h post-infection and stored at -80°C. Subsequently, fleas were mechanically disrupted using a FastPrep24 bead beater (MP Biomedicals, Santa Ana, California), as previously described [43]. Aliquots of triturated fleas were plated in BHI soft agar overlays, supplemented with 100 μg/ml carbenicillin and 10 μg/ml hemin, to determine the prevalence of infection and average CFU per infected flea. Fleas that ingested fluorescent beads were not used for infection rate or CFU assays.

### Early-phase mass transmission

Mass transmission experiments were performed as described previously [6]. Briefly, 3 days following infection, ~200 fleas (approximately equal numbers of males and females) were allowed to feed for 90 minutes on sterile defibrinated rat blood in the artificial feeding system. After feeding, fleas were collected to determine how many had fed, and of those, how many had evidence of digestive tract obstruction (fresh red blood in the esophagus). Blood was removed from the feeding apparatus and the interior of the feeder was washed ten times with 3 ml PBS. The entire volume of blood, as well as resuspended material concentrated by centrifugation from the PBS wash step, were distributively plated on blood agar plates. The external surface of the mouse skin membrane was disinfected with ethanol, cut into small pieces, and homogenized using a bead beating apparatus (MP Biomedicals, Santa Ana, CA). Skin samples were concentrated by centrifugation and plated separately on blood agar. Fleas that ingested fluorescent beads were never used for EPT assays. Blood agar plates were incubated at 28° C for 48 h prior to colony counts.

### Flea imaging and microscopy

Fleas were placed in PBS on a glass microscope slide and dissected with a set of fine forceps. The flea exoskeleton was removed and a glass cover slip was placed over the top of the digestive tract. For scoring esophageal localization of *Y*. *pestis*, digestive tracts in which ~25% or more of the length of the esophagus contained GFP+ bacteria were considered positive. Digestive tracts in which the proventricular valve contained red material that obscured visualization of at least 50% of the proventricular spines in bright field images were counted as positive for accumulation of partially digested blood in the proventriculus. For presence or absence of oxyhemoglobin crystals, 8 fields (half at 20x and half at 40x magnification) of digestive debris that leaked out of the midgut were screened per flea, and if any rectangular, pyramidal, rhomboidal, or trapezoidal objects were observed, the flea was considered positive for hemoglobin crystals. Intact fleas were immobilized by placing them on ice for 5–10 minutes, transferred to a drop of PBS on a microscope slide, overlaid with a glass cover slip, and PBS was added beneath the coverslip to completely submerge the flea. After 1 minute, the fleas were observed for 3–5 minutes for proventricular contraction and midgut peristalsis. To determine the location of ingested fluorescent beads in the digestive tract, fleas were examined by fluorescence microscopy. Images and videos of flea digestive tracts, bacterial biofilms, and fluorescent bead-laden fleas were obtained using a Nikon Eclipse E800 microscope or a Nikon SMZ 1500 dissecting microscope. The B-2A and G-2E/C fluorescent filter sets (Nikon) were used to acquire images of fleas containing GFP+ bacteria and fluorescent beads. Pictures and videos were obtained with an Olympus DP72 camera and cellSens software.

### Statistical analysis

All analyses were performed using GraphPad Prism 7 (GraphPad Software Inc., La Jolla, Ca.). Statistical tests used and relevant *p* values are indicated in the figure legends.

### Ethics statement

All experiments involving animals were approved by the Rocky Mountain Laboratories, National Institute of Allergy and Infectious Diseases, National Institutes of Health Animal Care and Use Committee (protocols 16–011-E and 16–058) and were conducted in accordance with all National Institutes of Health guidelines.

## Supporting information

S1 Fig The presence of rat erythrocytes in an infectious blood meal is sufficient to induce PIER. Incidence of PIER in *X*. *cheopis* fleas 24 h after they had fed on defibrinated mouse blood, heparinized rat blood, washed rat RBCs in PBS, or lysed rat RBCs mixed with defibrinated mouse blood containing 4 x 10^8^–7.9 x 10^8^ CFU/ml *Y*. *pestis* KIM6+ (pAcGFP1). The cumulative mean and range of 2–3 independent experiments (n = 181–329 fleas) are shown. *p < 0.0001 compared to defibrinated mouse blood group by chi-square test with Bonferonni post-test.
(TIF)

S2 Fig Analysis of rat hemoglobin crystals. (A) Following hemolysis, crystals were isolated by centrifugation and resuspended in sterile PBS. (B) Absorption spectra of a 1:20 dilution of rat hemoglobin crystals. Samples were run in duplicate and data are the mean of 2 independent experiments. Scale bar = 10 μm.
(TIF)

S3 Fig *Y*. *pestis* agglutinates rat and mouse erythrocytes equivalently. Agglutination pattern of washed mouse or rat erythrocytes mixed with 2-fold serial dilutions of 5x10^7^ CFU/ml KIM6+ (pAcGFP1) *Y*. *pestis* cultured at 37°C and suspended in PBS. Assay is representative of 3 independent experiments with 2 technical replicates per rodent blood source. Complete hemagglutination (hazy pattern) is observable until the 1:16 bacterial dilution after which point only partial or no agglutination (bull’s eye pattern) is observed.
(TIF)

S1 Video Example of post-feeding digestive tract activity of fleas with PIER. 24 hours after ingesting rat blood containing ~8 x 10^8^ KIM6+ (pAcGFP1) *Y*. *pestis* CFU/ml, *X*. *cheopis* were scored for PIER. Fleas with PIER were immobilized by placing them on ice for 5–10 minutes. Fleas were then placed in PBS on a microscope slide and a coverslip was gently laid over the top of the flea. After placement of the coverslip, fleas sat at room temperature for ~1 minute and were visualized for the next 3–5 minutes for proventricular contractions and midgut peristalsis. Of 34 PIER^+^ fleas from 2 independent experiments, only one exhibited proventricular contractions. Midgut peristalsis was observed in 8 fleas, an example of which is shown in this video. The 15 second, real-time video shows an infected flea with a static proventricular valve but an active midgut.
(MP4)

S2 Video Example of post-feeding digestive tract activity of fleas without PIER. Fleas without PIER were examined as described in S1 Video. Of 26 fleas from two independent infections using mouse (n = 16) or rat blood (n = 10), 15 (57%) exhibited regular, rhythmic proventricular contractions. Of those fleas where proventricular contractions were observed, 10 (66%) were infected using mouse blood. The number of PIER^-^ fleas that exhibited proventricular contractions was significantly different from PIER^+^ fleas (p < 0.0001 by chi-squared test). The 10 second, real-time video shows normal proventricular contractions and midgut peristalsis in an infected flea without PIER.
(MP4)
