# Supplementary figures and images for: Infectious blood source alters early foregut infection and regurgitative transmission of Yersinia pestis by rodent fleas
Source: PLoS Pathog. 2018 Jan 22;14(1):e1006859. doi: 10.1371/journal.ppat.1006859 (PMC5794196; doi:10.1371/journal.ppat.1006859)

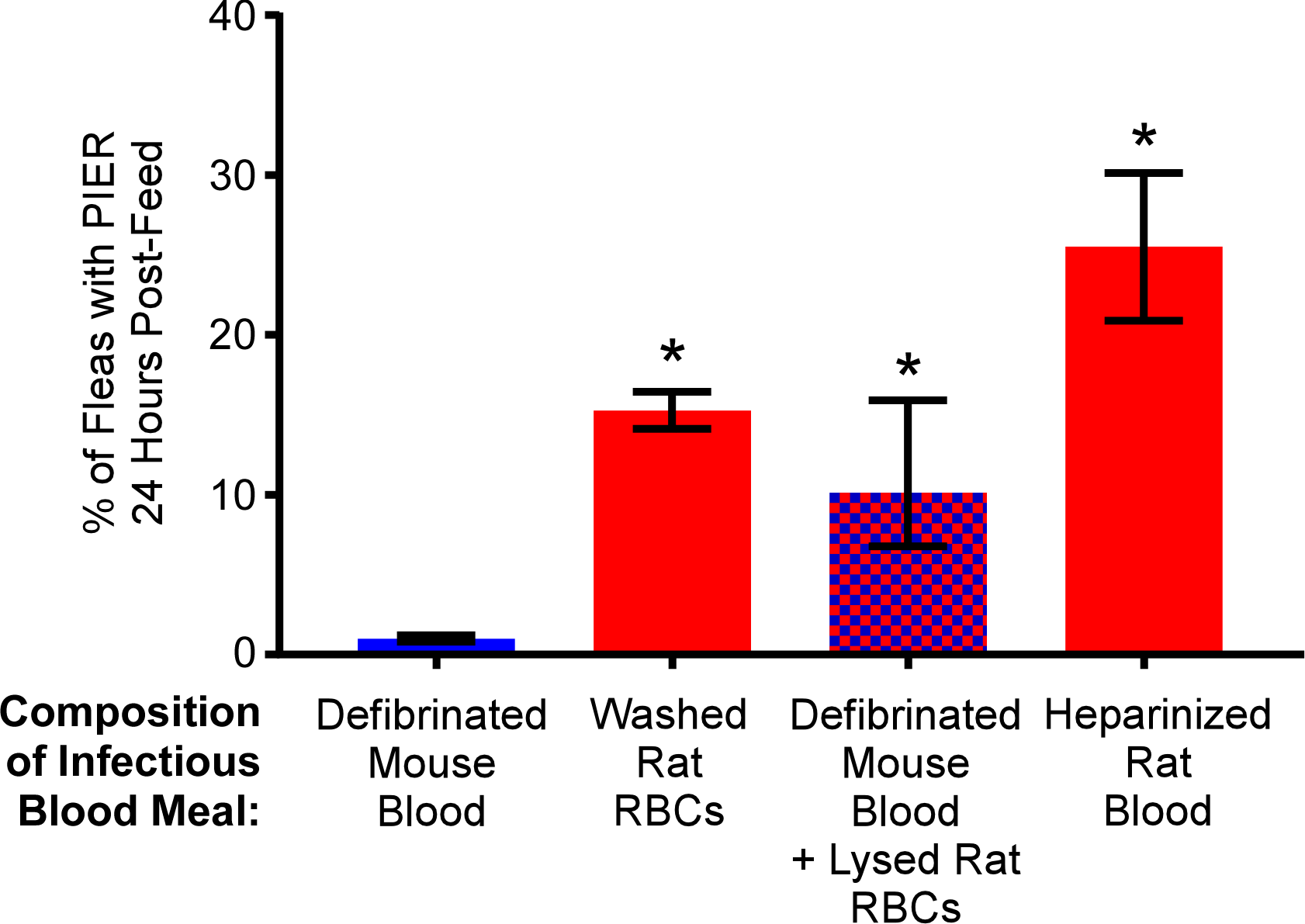

Supplement: S1 Fig — Incidence of PIER in X. cheopis fleas 24 h after they had fed on defibrinated mouse blood, heparinized rat blood, washed rat RBCs in PBS, or lysed rat RBCs mixed with defibrinated mouse blood containing 4 x 10^8^–7.9 x 10^8^ CFU/ml Y. pestis KIM6+ (pAcGFP1). The cumulative mean and range of 2–3 independent experiments (n = 181–329 fleas) are shown. *p < 0.0001 compared to defibrinated mouse blood group by chi-square test with Bonferonni post-test. (TIF) [file ppat.1006859.s001.tif]

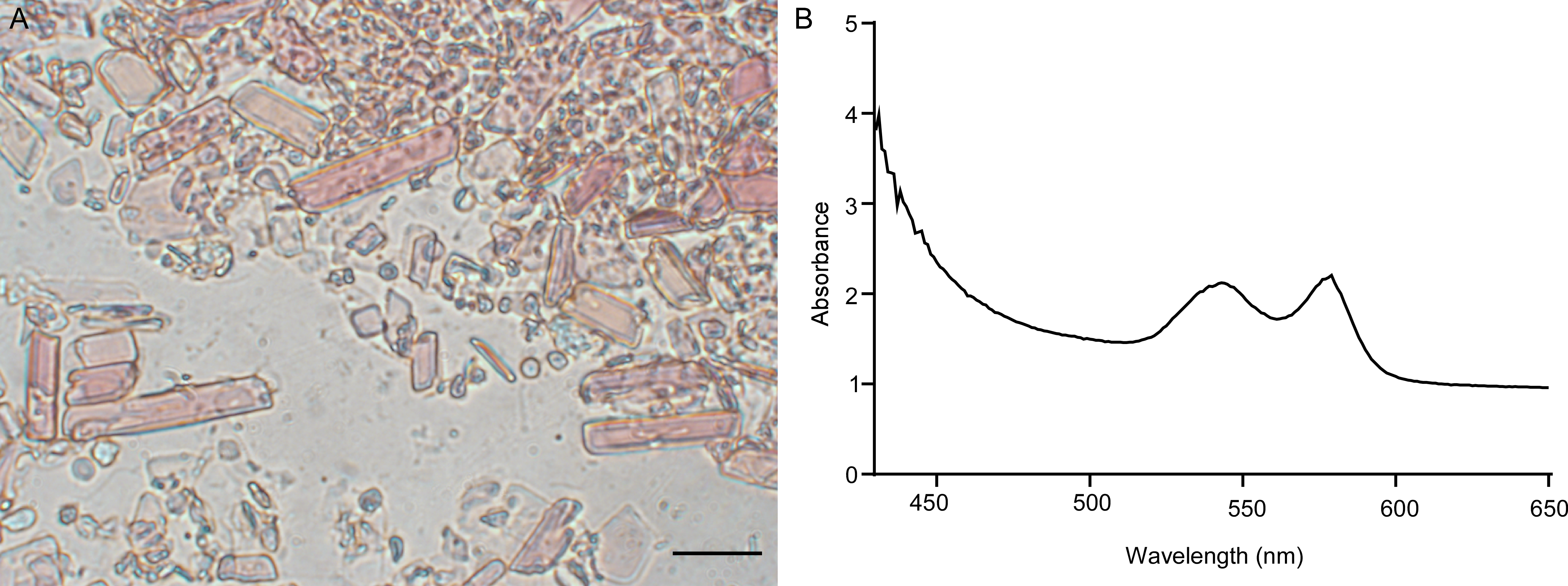

Supplement: S2 Fig — (A) Following hemolysis, crystals were isolated by centrifugation and resuspended in sterile PBS. (B) Absorption spectra of a 1:20 dilution of rat hemoglobin crystals. Samples were run in duplicate and data are the mean of 2 independent experiments. Scale bar = 10 μm. (TIF) [file ppat.1006859.s002.tif]

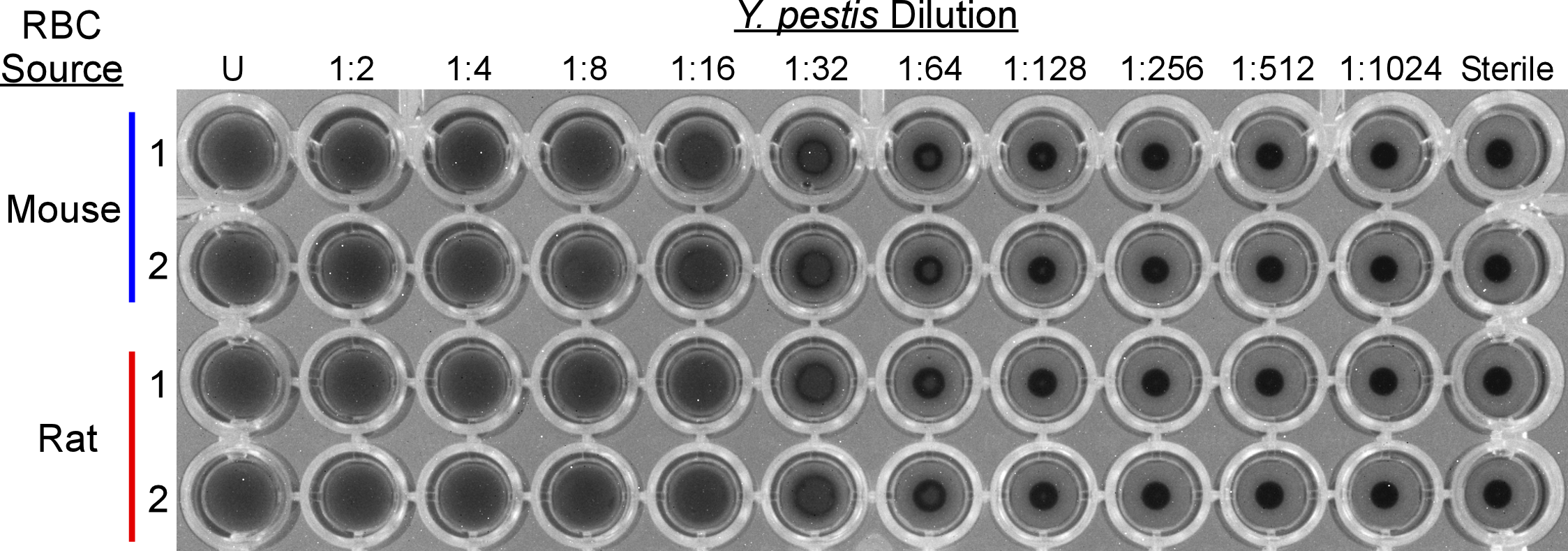

Supplement: S3 Fig — Agglutination pattern of washed mouse or rat erythrocytes mixed with 2-fold serial dilutions of 5x10^7^ CFU/ml KIM6+ (pAcGFP1) Y. pestis cultured at 37°C and suspended in PBS. Assay is representative of 3 independent experiments with 2 technical replicates per rodent blood source. Complete hemagglutination (hazy pattern) is observable until the 1:16 bacterial dilution after which point only partial or no agglutination (bull’s eye pattern) is observed. (TIF) [file ppat.1006859.s003.tif]
